# Relationships Between RNA Polymerase II Activity and Spt Elongation Factors to Spt^-^ Phenotype and Growth in *Saccharomyces cerevisiae*

**DOI:** 10.1534/g3.116.030346

**Published:** 2016-06-03

**Authors:** Ping Cui, Huiyan Jin, Manjula Ramya Vutukuru, Craig D. Kaplan

**Affiliations:** Department of Biochemistry and Biophysics, Texas A&M University, College Station, Texas 77843

**Keywords:** cryptic transcription, Ty1 element, transcription initiation, gene expression, transcription elongation

## Abstract

The interplay between adjacent transcription units can result in transcription-dependent alterations in chromatin structure or recruitment of factors that determine transcription outcomes, including the generation of intragenic or other cryptic transcripts derived from cryptic promoters. Mutations in a number of genes in *Saccharomyces cerevisiae* confer both cryptic intragenic transcription and the Suppressor of Ty (Spt^-^) phenotype for the *lys2-128*∂ allele of the *LYS2* gene. Mutants that suppress *lys2-128*∂ allow transcription from a normally inactive Ty1 ∂ promoter, conferring a *LYS^+^* phenotype. The arrangement of transcription units at *lys2-128*∂ is reminiscent of genes containing cryptic promoters within their open reading frames. We set out to examine the relationship between RNA Polymerase II (Pol II) activity, functions of Spt elongation factors, and cryptic transcription because of the previous observation that increased-activity Pol II alleles confer an Spt^-^ phenotype. We identify both cooperating and antagonistic genetic interactions between Pol II alleles and alleles of elongation factors *SPT4*, *SPT5*, and *SPT6*. We find that cryptic transcription at *FLO8* and *STE11* is distinct from that at *lys2-128*∂, though all show sensitivity to reduction in Pol II activity, especially the expression of *lys2-128*∂ found in Spt^-^ mutants. We determine that the *lys2-128*∂ Spt^-^ phenotypes for *spt6-1004* and increased activity *rpo21/rpb1* alleles each require transcription from the *LYS2* promoter. Furthermore, we identify the Ty1 transcription start site (TSS) within the ∂ element as the position of Spt^-^ transcription in tested Spt^-^ mutants.

It has become increasingly clear that eukaryotic genomes are extensively and pervasively transcribed outside of coding regions. Furthermore, beyond pervasive transcription, which in many cases is suppressed at the RNA level by RNA processing and decay pathways (Jensen *et al.* 2013; Libri 2015), genomes encode the potential for additional transcription in the form of cryptic promoters and transcription units (Hennig and Fischer 2013; Smolle and Workman 2013). Some cryptic transcription units may have the potential to be regulated (expressed under certain conditions) (Cheung *et al.* 2008), while others may become apparent upon disruption of chromatin structure or nuclear pathways that suppress them (Kaplan *et al.* 2003; Rando and Winston 2012). A complete understanding of gene regulation will require incorporation of knowledge about the relationships between adjacent transcription units and how they influence each other. To this end, *Saccharomyces cerevisiae* has been a powerful system for understanding the pervasive transcription, cryptic transcription, and transcriptional interference that can occur among proximally located transcription units.

Now-classic genetic screens in *S. cerevisiae* have identified a number of general or widely used transcription factors through alterations in locus-specific gene expression. A paradigm for these types of genetic analyses has been the *S*u*p*pressor of *T*y (Spt) screens, which leveraged transposable-element mediated changes in gene expression at particular auxotrophic gene loci for the identification of genetic suppressors of transcription defects (Winston 1992). Spt screens were able to identify a conserved and essential general transcription factor (*SPT15*, encoding TBP) (Eisenmann *et al.* 1989; Hahn *et al.* 1989), members of transcriptional coactivator complexes (a number of SAGA subunits, a subunit of Mediator) (Winston *et al.* 1984, 1987; Fassler and Winston 1988, 1989; Gansheroff *et al.* 1995; Marcus *et al.* 1996; Roberts and Winston 1996; Grant *et al.* 1997), histones and histone chaperones (*HTA1-HTB1*, *SPT6*, and *SPT16*)(Clark-Adams and Winston 1987; Clark-Adams *et al.* 1988; Fassler and Winston 1988; Malone *et al.* 1991; Rowley *et al.* 1991), conserved elongation factors (*SPT4* and *SPT5*), and factors regulating histone gene expression (*HIR2*, *SPT10*, and *SPT21*)(Natsoulis *et al.* 1991; Sherwood and Osley 1991). While not found in Spt screens, mutations in a number of other factors have the ability to confer Spt^-^ phenotypes, indicating both the sensitivity and possible complexity of these phenotypes.

The major shared characteristic of disrupted genetic loci used for Spt^-^ screens is that a Ty1 retroelement insertion disrupts normal gene expression while creating a compound transcription unit (Winston *et al.* 1984, 1987; Winston 1992). Compound transcription units are created because Ty1 elements contain both Ty1 promoter and terminator regions within the inserted Ty1 LTR DNA (known as a ∂ element). For one particular Spt allele, *lys2-128*∂ (the allele relevant to the work presented here), the Ty1 ∂ element is inserted within the *LYS2* ORF, causing premature termination of *LYS2* transcription, presumably at the Ty1 terminator, but also placing a Ty1 ∂ promoter in the path of *LYS2* transcription (Winston *et al.* 1987). Spt^-^ mutants that suppress *lys2-128*∂ allow transcription from within the Ty1 ∂ element to produce a 5′ truncated but functional *LYS2* mRNA (Clark-Adams and Winston 1987; Swanson *et al.* 1991; Malone *et al.* 1993). This suppression results in the Lys^+^ phenotype for Spt^-^ suppressor mutants of *lys2-128*∂, whereas WT *lys2-128*∂ strains are Lys^-^. This arrangement likely sensitizes Ty1 ∂ initiation to *LYS2* transcription and transcription-mediated chromatin control. Naturally occurring compound transcription units containing transcribed promoter elements have been found in the yeast genome and many of these exhibit modulation by many factors (Martens *et al.* 2004, 2005; Hainer *et al.* 2011; Bird *et al.* 2006). Furthermore, widespread antisense transcription is being revealed as a mechanism for shaping gene regulation in a number of ways, including transcription over promoters (Pelechano *et al.* 2013; Castelnuovo *et al.* 2014; Nguyen *et al.* 2014; Murray *et al.* 2015).

Extensive molecular characterization of intragenic cryptic transcription and control of the yeast gene *SER3* repression by transcription and transcription-dependent chromatin control (by the *SRG1* noncoding RNA that overlaps the *SER3* promoter) have revealed principles by which transcription units may regulate other transcription units. In the cases of *SER3* and intragenic cryptic promoters, transcription over these regions (from *SRG1* for *SER3* and from regular gene promoters for cryptic promoters) establishes a chromatin structure that is refractory to initiation of the transcribed promoter (Kaplan *et al.* 2003; Martens *et al.* 2004, 2005; Hainer *et al.* 2011; Kaplan 2013). In the case of *SER3*, transcription is required for establishment of the inhibitory chromatin structure, but for some cryptic promoters, transcription under aberrant conditions is required to disrupt chromatin structure and allow cryptic transcription (Cheung *et al.* 2008). These relationships allow factors to participate in repression of cryptic or transcribed promoters that otherwise function in activation for other promoters. Relatedly, Spt transcription elongation factors (*e.g.*, Spt4/Spt5, Spt6, and Spt16) are recruited almost universally to transcribed chromatin via Pol II (Andrulis *et al.* 2000; Kaplan *et al.* 2000; Saunders *et al.* 2003; Mayer *et al.* 2010), and have many attributes of positive factors. In contrast, initial genetic analyses of their function at Spt reporter genes indicated negative functions, because wild-type (WT) alleles of these genes are required to suppress cryptic Spt promoters.

Alteration of Pol II activity levels through active site mutations can have wide ranging effects on gene expression, including transcription start site selection, mRNA processing, termination, and overall transcription levels (reviewed in Kaplan 2013). Interestingly, a class of Pol II active site mutant that exhibits increased catalytic activity confers the Spt^-^ phenotype for *lys2-128*∂ (Kaplan *et al.* 2008, 2012). These results suggested that lys2-128∂ Spt^-^ phenotypes might be sensitive to Pol II activity, and that altered Pol II activity might contribute to cryptic transcription. We also were led to wonder how the Spt^-^ phenotypes for Spt elongation factors relate to their functions during Pol II transcription. A direct relationship between their functions during Pol II transcription and regulation of Pol II activity might predict that some Spt^-^ phenotypes could be a manifestation of loss of negative roles in Pol II activity (because increased Pol II activity correlates with the Spt^-^ phenotype). Alternatively, phenotypes for distinct Spt factors may have different origins. To address these questions, we examined genetic interactions between a panel of Pol II alleles with known consequences for Pol II activity and alleles in *spt4*, *spt5*, and *spt6*, while probing the relationship between Pol II and cryptic transcription for *FLO8*, *STE11*, and *lys2-128*∂.

## Materials and Methods

### Yeast strains, plasmids, and media

Yeast strains and plasmids used in this study are found in Supplemental Material, Table S1 and Table S2, respectively. Please refer to Table S2 or (Jin and Kaplan 2014) for a note on the *rpb1-N1082S* (*+T1161R*) plasmid used in these studies. Yeast media were prepared according to standard protocols (Amberg *et al.* 2005), with modifications described in Kaplan *et al.* (2012). All yeast strains are isogenic to a *GAL2*^+^ derivative of S288C (Winston *et al.* 1995). Yeast media for the growth of cultures for RNA isolation for examination of *lys2-128*∂ transcription were either YPD, SC-Leu, or “Synthetic Defined” (SD), supplemented with appropriate amino acids/nucleobases (including lysine), as noted. Strains for the examination of *lys2-128*∂ in minimal media were amplified overnight in YPD, washed in sterile H_2_O, and aliquots resuspended in SC-Leu or supplemented SD and grown for around three doublings prior to harvesting. All other media for plate phenotyping and growth are as described in Jin and Kaplan (2014).

### Heat maps for genetic interaction phenotypes

Phenotyping was as described in Jin and Kaplan (2014). Heatmaps and semiquantitative phenotype scoring were done as in Jin and Kaplan (2014). Briefly, growth on each medium was quantified using a 0–5 scoring scale (0 = no growth, 5 = WT growth for media except YPRafGal and SC-Lys; 0 = WT growth, 5 = growth of the mutant with maximum growth on the relevant YPRafGal or SC-Lys plate). Synthetic growth defects in Pol II and Spt^-^ mutants on YPD and SC-Leu were calculated by subtracting the mutant growth score by the WT growth score; growth differences on phenotype medium were calculated by normalizing the growth difference on the phenotype plate to the corresponding control medium to obtain the net difference caused by phenotypes. For determination of suppression of *spt5-242* by Pol II mutants at different temperatures, all mutants on YPD at different temperatures were normalized to WT on each corresponding plate by subtraction. Calculated score difference tables were turned into heatmaps using GENE-E (http://www.broadinstitute.org/cancer/software/GENE-E/index.html).

### RNA isolation and northern blotting

RNA isolation and northern blotting were as previously described (Kaplan *et al.* 2012). Briefly, RNA isolation followed the method of Schmitt *et al.* (1990). Initial northern blotting utilized formaldehyde-containing RNA gels as in Kaplan *et al.* (2012), while later gels were run using BPTE buffer with no formaldehyde (Sambrook and Russell 2001). For these later gels, RNA samples were treated with glyoxal loading dye (Ambion) following the manufacturer’s instructions. Blotting was as in Kaplan *et al.* (2012). Northern probes were labeled using random priming (Decaprime II Random Priming kit, Ambion) following the manufacturer’s instructions. The *SED1* probe was first amplified from yeast genomic DNA by PCR using oligonucleotide primers CKO468 and CKO469 (oligo sequences provided in Table S3). The *LYS2* 5′ end probe was amplified from *LYS2^+^* genomic DNA (*i.e.*, not *lys2-128*∂) using primers CKO551 and CKO552 so that the probe might hybridize to transcripts arising from the *LYS2* promoter and *lys2-128*∂, as long as the respective transcripts contained sequence derived from within ∼2200 bp from the 5′ end of *LYS2*. Since the ∂ element of *lys2-128*∂ is inserted after position +159 [Figure S2, reported as +158 in Fassler and Winston (1988)] from the *LYS2* ATG, this probe will detect transcripts from upstream of the ∂ and those emanating from the ∂ into *LYS2*.

### 5′ rapid amplification of cDNA ends (RACE), 3′ RACE, and primer extension analysis of lys2-128∂ transcripts

Primer extension followed the method of Ranish and Hahn (1991) (http://research.fhcrc.org/content/dam/stripe/hahn/methods/mol_biol/Primer%20extension%20yeast%20RNA.pdf) exactly, with volume modifications described in Kaplan *et al.* (2012). CKO1569 was used to detect *lys2-128*∂ RNAs. The GeneRacer RACE kit (Life Technologies) was used for 5′ RACE following the manufacturer’s instructions, using oligo CKO1569 for initial amplification of *lys2-128*∂ cDNAs, and CKO1585 for nested amplification. 3′ RACE was performed using Illustra Ready-to-Go cDNA beads (GE Healthcare Life Sciences). Oligo-dT plus linker sequence (CKO099) was used for first strand synthesis per the manufacturer’s instructions. Initial 3′ RACE PCR was done with CKO843 *LYS2* primer and CKO100 primer (to CKO099 linker) (35 cycles). Nested PCR was with primers CKO844 (*LYS2* primer) and CKO101 primer (to CKO099 linker) (35 cycles). RACE cDNAs were cloned by TOPO-Blunt cloning (Life Technologies) and their inserts sequenced.

### Data availability

Yeast strains and plasmids described in this work are stored in the Kaplan lab strain collections and are available upon request. Supplemental materials for this work are as follows: Figure S1, Figure S2, and Figure S3 and their associated legends, Table S1 of yeast strains used, Table S2 of plasmids, and Table S3 of oligonucleotide sequences (see also File S1).

## Results

### Extensive allele-specific genetic interactions between Pol II alleles and SPT elongation factor mutant alleles

We wished to understand the relationship between altered Pol II catalytic activity and the Spt^-^ phenotype. To explore this relationship, we examined genetic interactions between Pol II alleles with altered catalytic properties and alleles of Spt elongation factors that also confer Spt^-^ phenotypes. We assessed genetic interactions between a panel of *rpo21/rpb1* alleles, most of which encode substitutions in the Pol II active site trigger loop, and alleles of Spt elongation factors *SPT4*, *SPT5*, and *SPT6*. Pol II trigger loop substitutions confer a range of catalytic activities *in vitro* and in many cases show allele-specific genetic interactions that correlate with the nature of the activity defects (Braberg *et al.* 2013). For example, genetic interactions can be positive/epistatic or negative depending on Pol II allele strength and whether the Pol II allele confers increased or decreased catalytic activity. We term mutants that increase Pol II catalytic activity (determined by *in vitro* elongation rate) as “gain of function” (GOF) and those that decrease catalytic activity as “loss of function” (LOF).

In order to assess genetic interactions, we utilized yeast strains containing a deletion in the largest subunit of Pol II, *rpo21*∆ (complemented by *RPO21/RPB1* expressed from its native promoter on a *CEN* plasmid), and the following *SPT* gene alleles: *spt4*∆, *spt5-194* [encoding E338K (Guo *et al.* 2008)], *spt5-242* (encoding A268V, G. Hartzog, personal communication), and *spt6-1004* [encoding a deletion of residues 931–994, replaced by a short linker (Kaplan 2003; Kaplan *et al.* 2005)]. We transformed each of these strains with a series of plasmids encoding Pol II alleles in *RPO21/RPB1*. In the course of yeast transformation experiments, we noted that the presence of certain *rpo21/rpb1* mutant plasmids conferred extremely slow growth on transformants dependent on the presence of *SPT* gene alleles (most obvious for *spt6-1004*, Figure S1). For *spt6-1004*, this phenomenon occurs with both reduced activity (loss of function or LOF) and increased activity (gain of function or GOF) Pol II mutants, but is stronger for LOF alleles. Furthermore, this uncovering of dominant *rpo21/rpb1* phenotypes extends to lethal *rpo21/rpb1* alleles encoding presumed extreme LOF and GOF variants, suggesting that these variants are assembled into Pol II complexes that can functionally interfere with WT subunit function.

**Figure 1:**
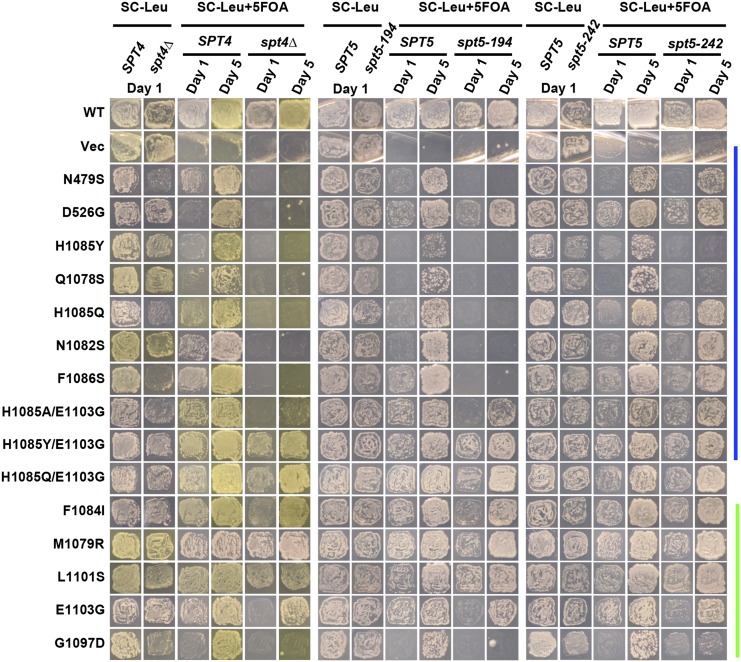
Allele-specific synthetic lethality between Pol II activity mutants and alleles of *SPT4* and *SPT5*. Yeast strains containing mutants of *SPT4* or *SPT5* and *rpo21*∆ were transformed with *LEU2*-marked *CEN* plasmids containing *rpo21/rpb1* alleles. Transformants were patched and replica-plated to media allowing *rpo21/rpb1* complementation by a *URA3*-marked *CEN RPO21/RPB1* plasmid (SC-Leu) or media toxic to cells maintaining the *URA3*-marked *CEN RPO21/RPB1* plasmid (SC-Leu+5FOA). Resulting growth of replica-plated patches was documented after 1 and 5 d of growth. Color bars indicate genetically predicted/known reduction of function (blue) or increase of function (green) *rpo21/rpb1* alleles. H1085Q/E1103G has mixed LOF/GOF properties based on genetic assays. GOF, gain of function; LOF, loss of function; Vec, vector only; WT, wild-type.

**Figure 2:**
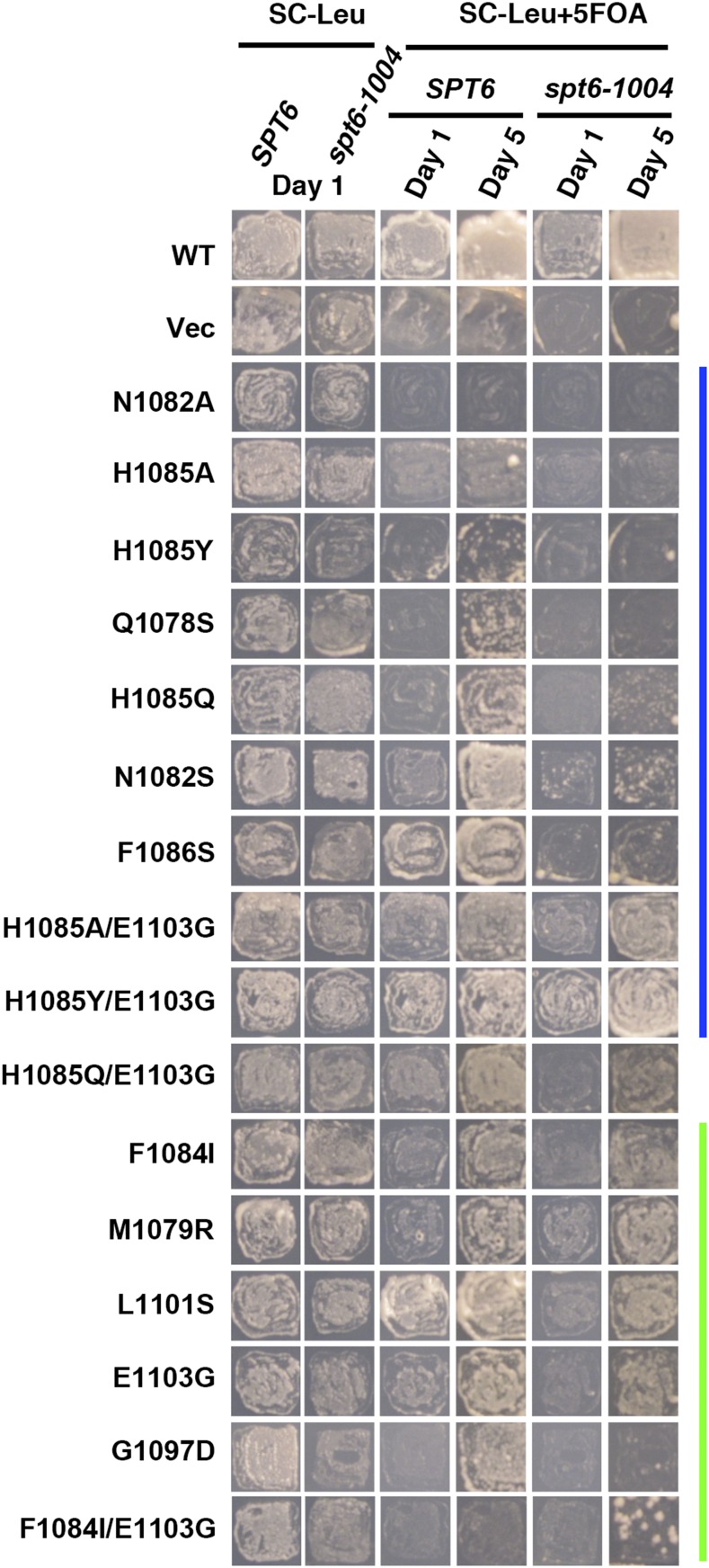
Allele-specific synthetic lethality between Pol II activity mutants and the *SPT6* allele, *spt6-1004*. Yeast strain containing *spt6-1004* and *rpo21*∆ was transformed with *LEU2*-marked *CEN* plasmids containing *rpo21/rpb1* alleles. Transformants were patched and replica-plated to media allowing *rpo21/rpb1* complementation by a *URA3*-marked *CEN RPO21/RPB1* plasmid (SC-Leu) or media toxic to cells maintaining the *URA3*-marked *CEN RPO21/RPB1* plasmid (SC-Leu 5FOA). Resulting growth of replica-plated patches was documented after 1 and 5 d of growth. Color bars indicate genetically predicted/known reduction of function (blue) or increase of function (green) *rpo21/rpb1* alleles. Vec, vector only; WT, wild-type.

**Figure 3:**
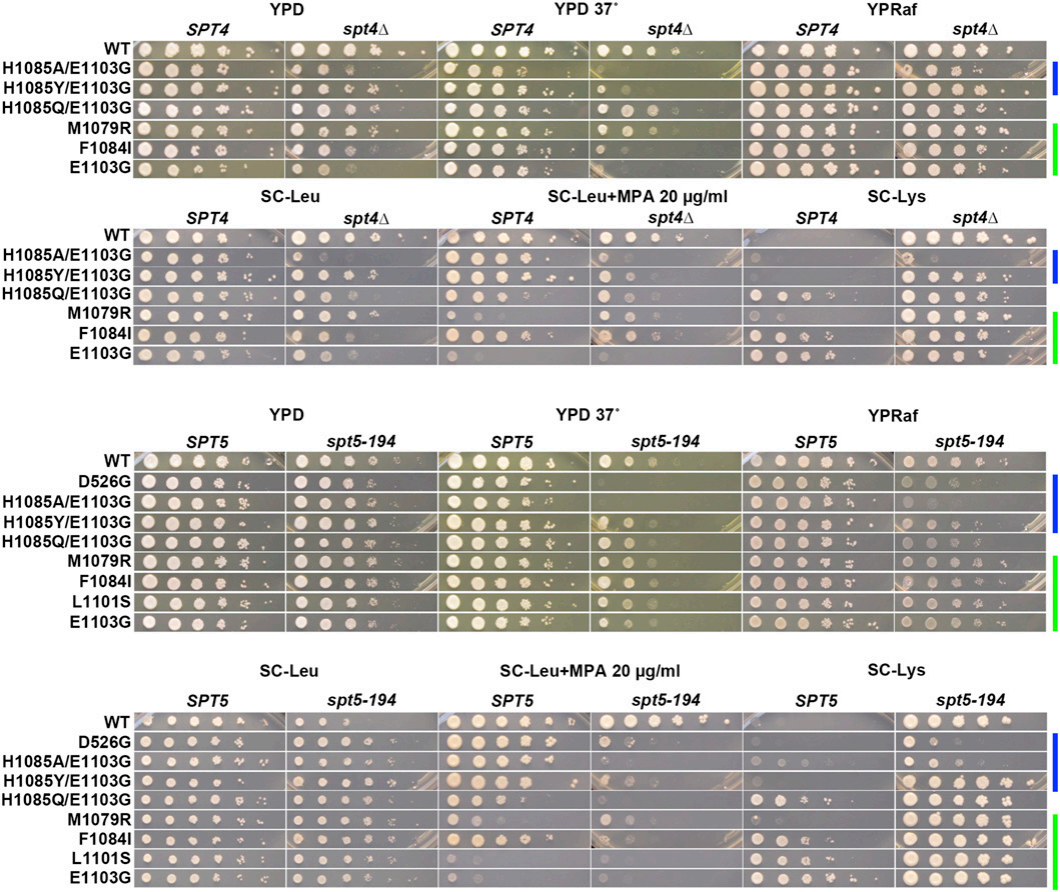
Conditional genetic interactions between Pol II activity mutants and *spt4*∆ and *spt5-194*. Phenotypes of viable double mutants between *spt4*∆ or *spt5-194* combined with *rpo21/rpb1* alleles (see *Materials and Methods*). Serial dilutions (10-fold) of control or double mutant strains spotted on various media for phenotyping of genetic interactions (general growth, temperature sensitivity, Spt^-^, MPA^S^, and Gal^R^ phenotypes). Color bars indicate genetically predicted/known reduction of function (blue) or increase of function (green) *rpo21/rpb1* alleles. Gal, galactose; MPA, mycophenolic acid; WT, wild-type; YPD, yeast extract peptone dextrose.

**Figure 4:**
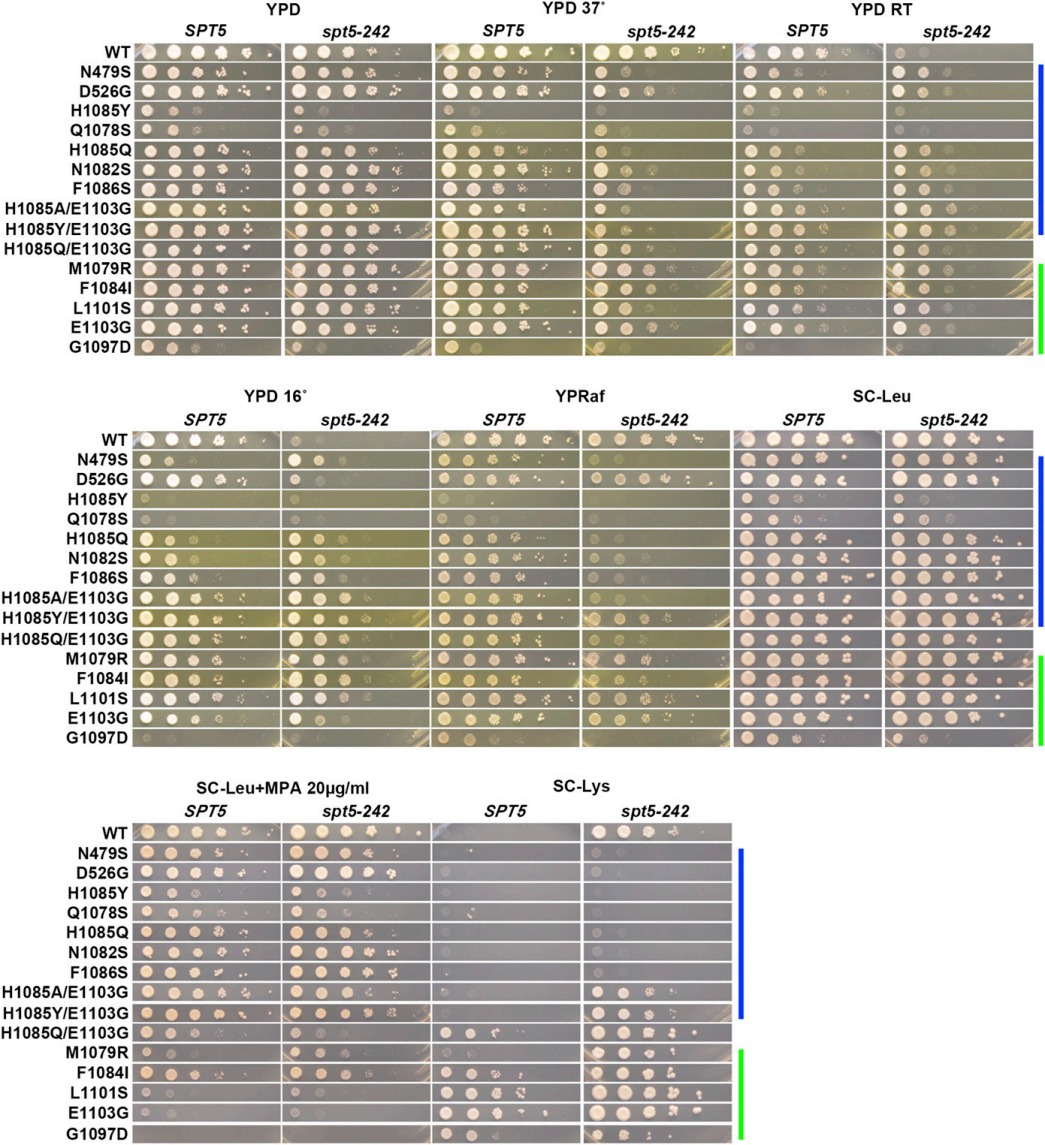
Conditional genetic interactions between Pol II activity mutants and *spt5-242*. Phenotypes of viable double mutants between *spt5-242* combined with *rpo21/rpb1* alleles (see *Materials and Methods*). Serial dilutions (10-fold) of control or double mutant strains spotted on various medial for phenotyping of genetic interactions (general growth, temperature sensitivity, Spt^-^, MPA^S^, and Gal^R^ phenotypes). Color bars indicate genetically predicted/known reduction of function (blue) or increase of function (green) *rpo21/rpb1* alleles. Raf, raffinose; Gal, galactose; MPA, mycophenolic acid; WT, wild-type; YPD, yeast extract peptone dextrose.

**Figure 5:**
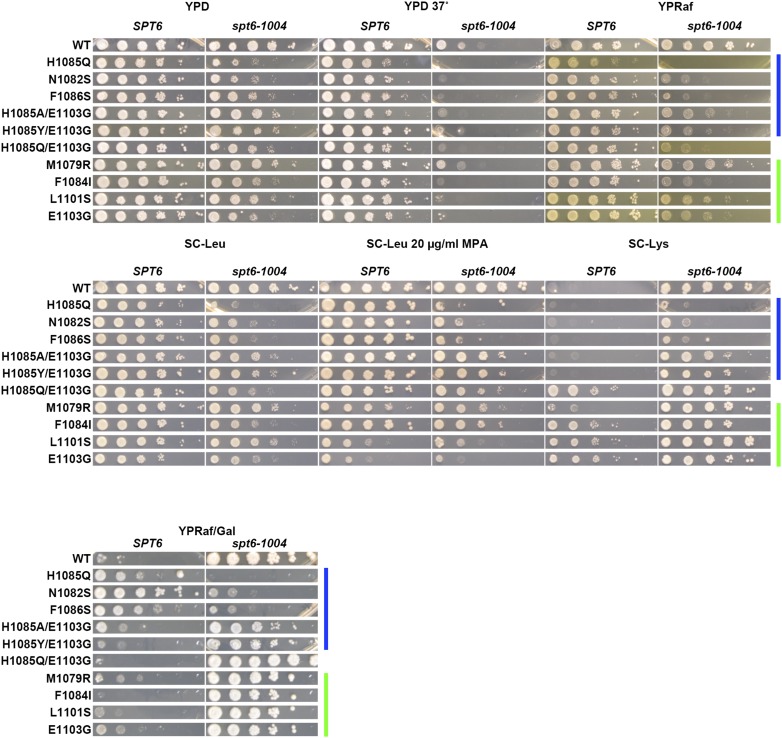
Conditional genetic interactions between Pol II activity mutants and *spt6-1004*. Phenotypes of viable double mutants between *spt6-1004* combined with *rpo21/rpb1* alleles (see *Materials and Methods*). Serial dilutions (10-fold) of control or double mutant strains spotted on various medial for phenotyping of genetic interactions (general growth, temperature sensitivity, Spt^-^, MPA^S^, and Gal^R^ phenotypes). Color bars indicate genetically predicted/known reduction of function (blue) or increase of function (green) *rpo21/rpb1* alleles. Raf, raffinose; Gal, galactose; MPA, mycophenolic acid; WT, wild-type; YPD, yeast extract peptone dextrose.

**Figure 6:**
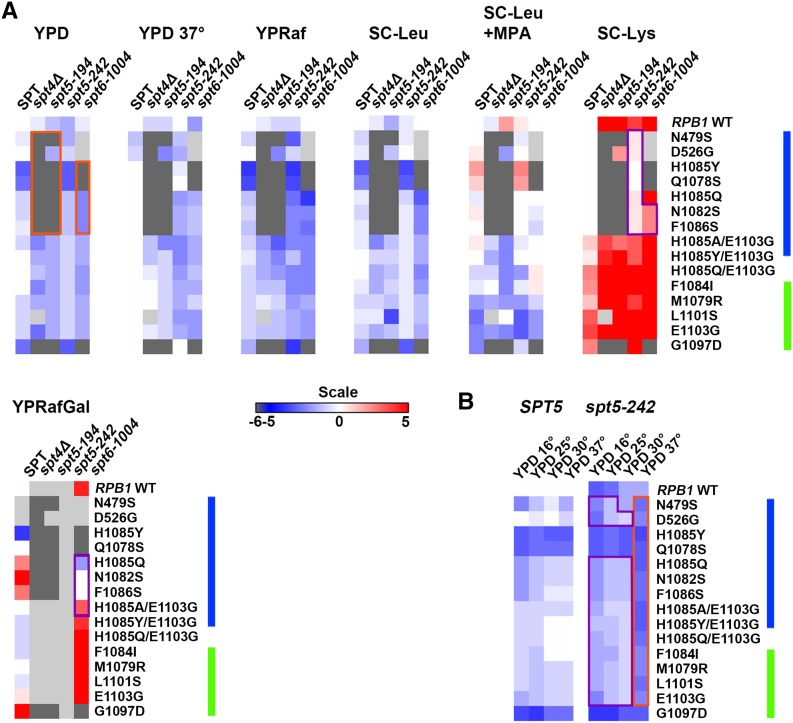
Summary of genetic interactions between Pol II activity mutants and Spt elongation factor alleles. Phenotypes of viable Spt-Pol II double mutants are shown as a heatmap with qualitative determinations of growth defects for the relevant media. Dark gray indicates inviable double mutants. Light gray indicates conditions/strains not tested. For YPD, YPD 37°, YPRaf, and SC-Leu media, single and double mutant growth levels were normalized to WT. Decreased growth relative to WT is shown in shades of blue. Red indicates increased growth relative to WT. For SC-Leu+MPA, growth differences were normalized to those on SC-Leu to account for general effects on growth *vs.* those specific to MPA treatment. Similarly, YPRafGal was normalized to defects on YPD, and SC-Lys to those on SC-Leu. See *Materials and Methods* for additional explanation of heatmaps. (A) Interactions between Spt gene alleles and Pol II mutants. Orange and purple outlines indicate examples of clusters of allele-specific interactions. (B) Temperature-dependent genetic interactions between *spt5-242* and Pol II alleles. Purple outline highlights suppression of *spt5-242* at lower temperatures (but apparent at 30°). Orange outline highlights decreased growth at 37° for double mutants. Raf, raffinose; Gal, galactose; MPA, mycophenolic acid; WT, wild-type; YPD, yeast extract peptone dextrose.

To reveal genetic interactions between Pol II alleles in the haploid state and *SPT* genes, we performed plasmid shuffling to select for strains having lost the WT *RPO21/RPB1* plasmid while retaining an *rpo21/rpb1* plasmid. Viability of strains upon plasmid shuffling was assayed by replica-plating to 5-FOA medium (Boeke *et al.* 1987) and is shown for *spt4*∆ and *spt5* alleles (Figure 1) and *spt6-1004* (Figure 2). Viable double mutant strains and single mutant and WT controls were further phenotyped for genetic interactions affecting growth under a number of conditions. These conditions included suppression or enhancement of growth phenotypes detected by reporter alleles, such as the Spt^-^ reporter *lys2-128*∂ (Simchen *et al.* 1984) or the transcription interference reporter *gal10*∆*56* (Kaplan *et al.* 2005), or those relating to gene-specific transcription defects, such as expression of *IMD2*, detected by sensitivity to mycophenolic acid (MPA) (Shaw and Reines 2000; Jenks *et al.* 2008; Kuehner and Brow 2008) [reviewed in Kaplan (2013)]. Results are shown for *spt4*∆ and *spt5-194* (Figure 3), *spt5-242* (Figure 4), and *spt6-1004* (Figure 5). Genetic interaction results are summarized in the form of heat maps and compiled in Figure 6.

We observed extensive allele-specific genetic interactions between Pol II alleles and *SPT* elongation factor alleles. First, we observed synthetic lethality and synthetic sickness much more commonly between Pol II LOF alleles and *spt4*∆, *spt5-194*, or *spt6-1004* than between Pol II GOF alleles and the same (Figures 1, Figure 2, Figure 3, Figure 4, Figure 5, and Figure 6). The similar responses of Pol II alleles to *spt4*∆ and *spt5-194* were expected based on previous experiments indicating defects in Spt4 association with Spt5 in *spt5-194* (Guo *et al.* 2008). The overall strength of interactions with Pol II LOF alleles appears to be *spt4*∆ *> spt5-194 > spt6-1004*, judging by decreasing synthetic lethality (summarized in Figure 6). Conversely, *spt4*∆ and *spt6-1004* show synthetic growth defects with a number of GOF Pol II alleles, as evidenced by reduced growth on rich (YPD) or defined (SC-Leu) media, that were not observed for combinations with *spt5-194*. For *spt5-194*, enhancement of MPA sensitivity was apparent for both presumed LOF Pol II alleles (D526G substitution outside the TL and compound TL alleles) and for presumed GOF alleles (M1079R, F1084I) (Figure 3 and Figure 6). Examination of conditional growth defects shows that *spt5-194* and Pol II double mutants can become MPA sensitive, even for combinations where neither single mutant itself was strongly MPA sensitive (Figure 3 and Figure 6).

Interactions between Pol II mutants and *spt5-242* were even more complex. *spt5-242* was originally identified as a cold sensitive (Cs^-^) allele of *SPT5* (Hartzog *et al.* 1998). The *spt5-242* Cs^-^ phenotype can be suppressed by a number of mutants or conditions, including alleles of genes encoding subunits of the Paf1C elongation complex (Squazzo *et al.* 2002), histone subunits (Quan and Hartzog 2010), chromatin remodelers (Simic *et al.* 2003), reduced function Pol II alleles (*rpb2-10*), or those with unknown effects on catalysis (*rpb1-221* and *rpb1-244*) (Hartzog *et al.* 1998), or the nucleotide-depleting drug 6-azauracil (Hartzog *et al.* 1998). The molecular defects of *spt5-242* in gene expression are poorly understood, though it confers apparent slow Pol II elongation *in vivo* (Quan and Hartzog 2010). The *spt5-242* defect has been proposed to relate to kinetic uncoupling between transcription and other elongation events leading to cold-sensitive growth (Hartzog *et al.* 1998). Slowing Pol II or relaxation of transcribed chromatin structure, specifically by loss of H3K4me or H3K36me modifications or factors that promote them (Paf1C factors), or function of its effectors (Rpd3S complex, Chd1), suppresses *spt5-242* cold sensitivity (Quan and Hartzog 2010). Similar to previous studies showing suppression of the *spt5-242* Cs^-^ phenotype by *rpb2-10* (Hartzog *et al.* 1998), an allele of *RPB2* causing elongation defects *in vivo* and *in vitro*, we observed suppression of *spt5-242* broadly in essentially all LOF Pol II alleles tested. Intriguingly, we also observed suppression of *spt5-242* by most GOF Pol II alleles tested (see *Discussion*). Furthermore, *spt5-242* confers a sensitivity to 37° for almost all Pol II alleles, whether LOF or GOF (Figure 4 and Figure 6). These results are consistent with *spt5-242* causing a continuum of defects from 15°–37° or having distinct defects at low and high temperatures that can be either suppressed or exacerbated by alteration in Pol II catalytic activity.

### Sensitivity of lys2-128∂ suppression to Pol II activity

**Figure 7:**
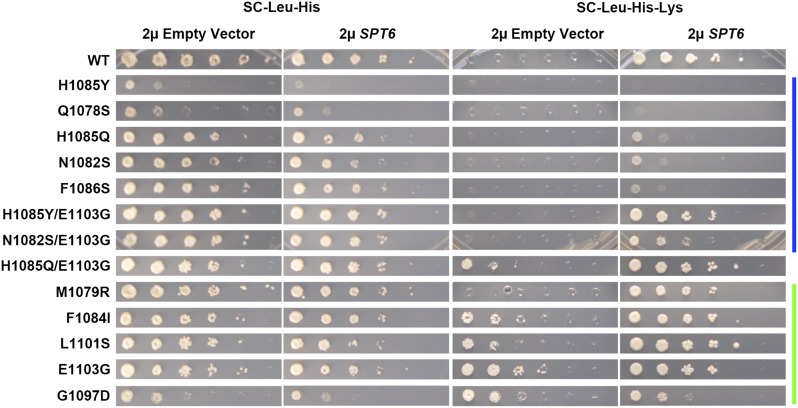
Pol II LOF alleles suppress the Spt^-^ phenotype of high copy *SPT6*. Strains containing *rpo21/rpb1* alleles were transformed by introduction of high copy (2µ) empty vector (pRS423) or *SPT6* (pRS423 *SPT6*) and general growth (SC-Leu-His medium) or Spt^-^ phenotypes (SC-Leu-His-Lys medium) were assessed. Color bars indicate genetically predicted/known reduction of function (blue) or increase of function (green) *rpo21/rpb1* alleles. LOF, loss of function; WT, wild-type.

Examination of suppression of *lys2-128*∂ (Spt^-^ phenotype) revealed complex relationships between Spt factors and Pol II alleles. A model that correlates Spt^-^ phenotypes with other underlying defects of Spt^-^ alleles predicts that Spt^-^ alleles of *SPT* elongation factor genes and Spt^-^ alleles of Pol II would show exacerbated phenotypes when combined, if their Spt^-^ defects arose from similar causes. This is not supported by the observed interactions. First, GOF Pol II alleles confer the Spt^-^ phenotype, yet there are much stronger genetic interactions between non-Spt^-^ LOF Pol II alleles and alleles of *SPT* elongation factor genes (discussed above). Second, while exacerbating other growth defects, LOF Pol II alleles suppressed Spt^-^ phenotypes of *spt4*∆ and *spt5-194* where the double mutants were viable. LOF Pol II alleles also suppressed the Spt^-^ phenotype of *spt5-242*, though both LOF and GOF Pol II alleles suppressed *spt5-242* cold sensitivity. Distinct from *spt4*∆ and *spt5* alleles, the Spt^-^ phenotype of *spt6-1004* was not strongly affected in *spt6-1004/*Pol II allele double mutants for GOF mutants. *spt6-1004/*LOF mutants were quite sick and appeared to be Spt^-^ when considering their overall growth defects. To further examine the relationship between Pol II activity and the Spt^-^ phenotype upon *SPT6* alteration, we examined the Spt^-^ phenotype conferred upon overexpression of *SPT6* (Figure 7). We asked if the Spt^-^ phenotype, due to expression of *SPT6* from a high copy plasmid, was sensitive to Pol II activity, and found that this Spt^-^ phenotype was also suppressed in LOF Pol II mutants. These results suggest that suppression of *lys2-128*∂ under a number of conditions is sensitive to Pol II activity.

### Cryptic intragenic transcription is distinct from transcription at lys2-128∂

**Figure 8:**
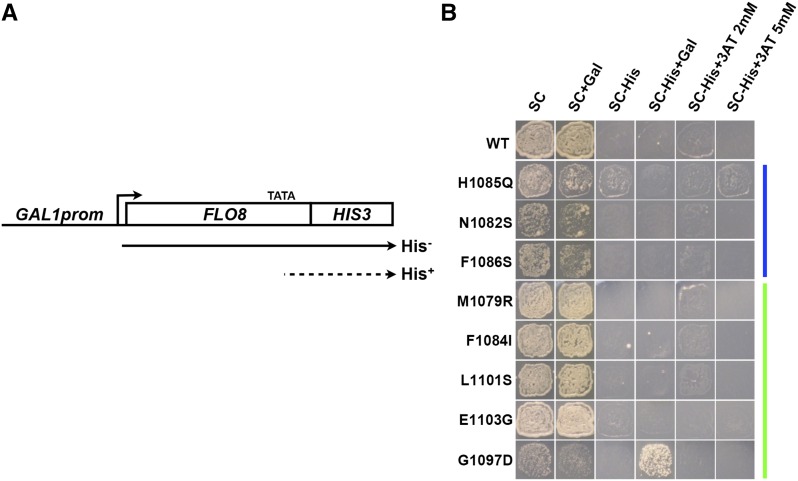
Derepression of the *FLO8* intragenic cryptic promoter occurs in an extreme Pol II allele, G1097D, and requires *FLO8* transcription. (A) Schematic of *GAL1promoter*::*FLO8*::*HIS3* construct allowing analysis of *HIS3*-dependent growth due to transcription from a cryptic *FLO8* intragenic promoter (denoted by “TATA”) driving *HIS3* expression. (B) Assay of *rpo21/rpb1* allele activation of the *FLO8* cryptic intragenic promoter dependent on activation of the *GAL1* promoter by presence of galactose in growth medium (+Gal). Gal, galactose; WT, wild-type.

**Figure 9:**
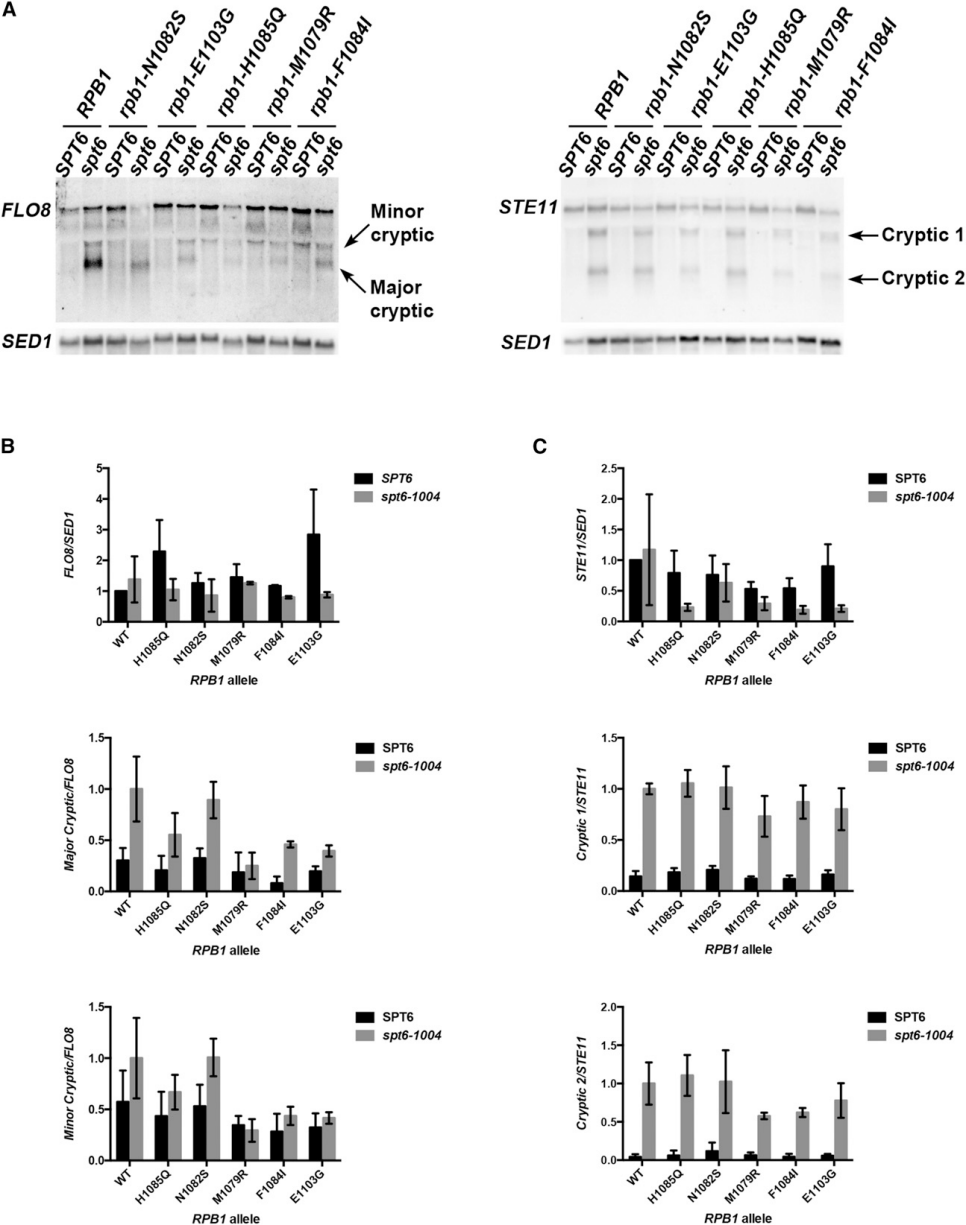
Expression levels of *FLO8* and *STE11* cryptic transcripts are sensitive to reduced Pol II activity mutants. (A) Northern blotting for *FLO8*-derived RNAs (left) or *STE11*-derived RNAs (right) in various mutant strains. Northern blotting for *SED1* provided a normalization control. *spt6* indicates presence of *spt6-1004* allele. (B) Quantification of mRNA levels for *FLO8*-derived transcripts (full-length, top; major cryptic, middle; and minor cryptic, bottom) normalized to *SED1*. Full-length *FLO8* expression was normalized relative to the level in WT. Levels of cryptic transcripts were normalized to full-length and then relative to those in *spt6-1004*. Error bars represent standard deviation of the mean for at least three independent biological replicates. (C) Quantification of mRNA levels for *STE11*-derived transcripts (full-length, top; cryptic 1, middle; and cryptic 2, bottom) normalized to *SED1*. Full-length *STE11* expression was normalized relative to the level in WT. Levels of cryptic transcripts were normalized relative to full-length then to those in *spt6-1004*. Error bars as in (B). WT, wild-type.

Given that Spt^-^ phenotypes are conferred by mutations in a number of factors that also confer cryptic transcription (Kaplan *et al.* 2003; Cheung *et al.* 2008), we asked if Pol II mutants conferred cryptic transcription. We reasoned that increasing Pol II activity through trigger loop substitutions, which correlates with an Spt^-^ phenotype, might allow suboptimal cryptic promoters to function. Supporting the idea that promoters might be sensitive to Pol II catalytic activity, transcription start site selection is altered upon alteration of the Pol II trigger loop, which directly influences Pol II catalysis (Kaplan *et al.* 2012; Braberg *et al.* 2013). Alternatively, altered Pol II elongation might confer chromatin defects that lead to both Spt^-^ and cryptic transcription phenotypes, as cryptic promoters are derepressed by disruptions in transcription-coupled chromatin assembly (reviewed in Smolle and Workman (2013). To assess cryptic transcription, we performed genetic and molecular characterization of transcription from a *FLO8* intragenic promoter reporter construct (Cheung *et al.* 2008) (Figure 8), as well as native *FLO8* and *STE11* cryptic promoters (Figure 9). We utilized a sensitive reporter for cryptic transcription developed by the Winston lab, wherein a *FLO8* intragenic promoter is fused to *HIS3* (*FLO8-HIS3*), with the native *FLO8* promoter replaced by the *GAL1* promoter (Cheung *et al.* 2008). This reporter allows cryptic transcription to be monitored by *HIS3* levels in the presence or absence of high levels of transcription from the *GAL1* promoter. A number of factors restrict expression from *FLO8-HIS3*, with many being revealed only under conditions of high *GAL1p*::*FLO8-HIS3* expression (Cheung *et al.* 2008). For Pol II mutants, only our most extreme GOF substitution (G1097D) conferred detectable *FLO8-HIS3* expression, as inferred through growth on medium lacking histidine, which requires *HIS3* gene expression. G1097D derepression of the *FLO8-HIS3* reporter also required galactose in the medium, presumably related to high-level expression of *FLO8-HIS3*. This result suggests that while it is possible for altered Pol II activity to promote cryptic expression, it is neither at a high level nor a widely distributed phenomenon for Pol II alleles when considering *FLO8-HIS3*. We then asked a converse question: is *spt6-1004* cryptic transcription sensitive to Pol II activity, given that Spt^-^ phenotypes of *spt* alleles can be suppressed by Pol II alleles. These observations raised the possibility that cryptic transcription might be sensitive to Pol II activity. Therefore, we examined native transcription for both *FLO8* and *STE11* in *SPT6* and *spt6-1004* backgrounds for Pol II catalytic mutants (Figure 9).

Northern blotting for *FLO8* and *STE11* showed previously identified cryptic transcripts in *spt6-1004* cells at a permissive temperature of 30°. *spt6-1004*/Pol II double mutants showed cryptic transcripts for both *FLO8* and *STE11*. Pol II mutants did not exhibit cryptic transcription on their own for either *FLO8* and *STE11*. Full-length *STE11*, but not *FLO8*, was generally reduced in *spt6-1004*/Pol II double mutants regardless of LOF or GOF character of the Pol II substitution, but cryptic mRNA levels relative to full-length mRNA levels were not affected. For *FLO8* cryptic transcripts, GOF Pol II alleles appear to reduce the ratio of cryptic transcripts relative to full-length in *spt6-1004* strains. These results suggest that apparent cryptic transcription is not overly sensitive to Pol II catalytic mutants, and is distinct from the sensitivity to reduced Pol II activity mutants apparent in their suppression of *lys2-128*∂.

### Transcription from the LYS2 promoter is both required for the Spt^-^ phenotype and can modulate genetic requirements for it

**Figure 10:**
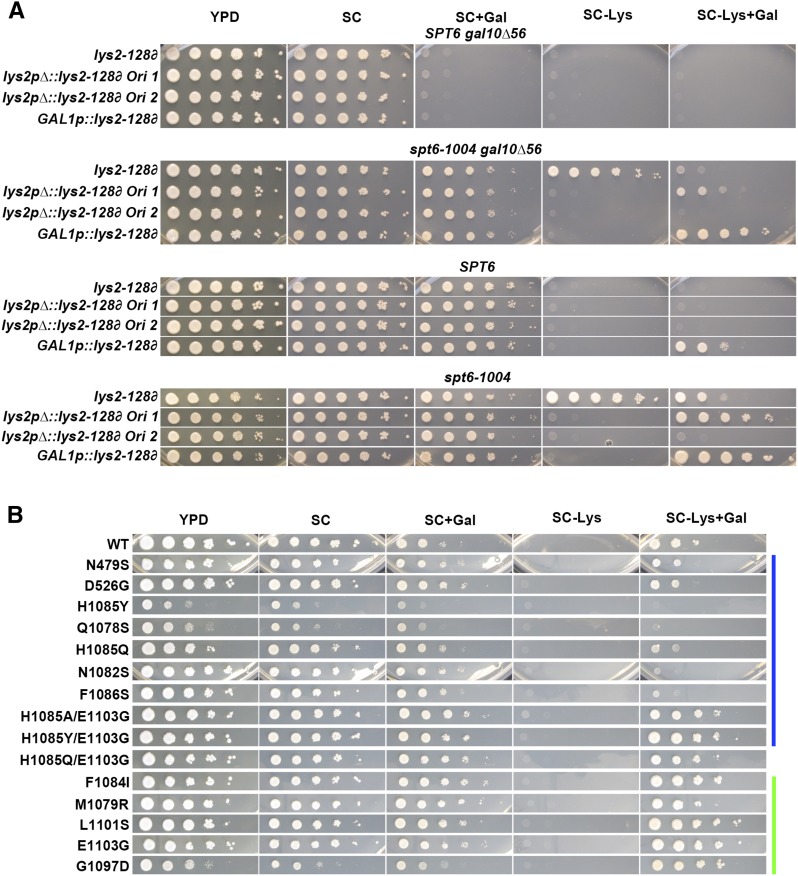
Suppression of *lys2-128*∂ by *spt6-1004* or *rpo21/rpb1* alleles requires upstream transcription and can be modulated by an ectopic promoter. (A) Analysis of *spt6-1004* effects on *lys2-128*∂ promoter derivatives. *lys2-128*∂ was modified in the promoter region for *LYS2* in *SPT6* and *spt6-1004* strains containing either *GAL10* or *gal10*∆*56*. Modifications included deletion of the *LYS2* promoter by homologous recombination-based insertion of a drug resistance cassette (in either orientation denoted “Ori 1” or “Ori 2”) or by replacement of the *LYS2* promoter with the *GAL1* promoter. Strains were assayed for the Spt^-^ phenotype on media containing galactose (+Gal). All other media used glucose as carbon source. Ten-fold serial dilutions of each strain spotted onto appropriate medium. (B) Analysis of *rpo21/rpb1* allele effects on *lys2-128*∂ promoter derivatives. The *LYS2* promoter was replaced with the *GAL1* promoter as in (B) for a strain allowing transformation with different *rpo21/rpb1* alleles. Analysis and conditions as in (B). Color bars indicate genetically predicted/known reduction of function (blue) or increase of function (green) *rpo21/rpb1* alleles. Gal, galactose; WT, wild-type; YPD, yeast extract peptone dextrose.

Experiments described above indicated that cryptic transcription and transcription leading to the Spt^-^ phenotype for *lys2-128*∂ have distinct properties. Notwithstanding this conclusion, we further explored commonalities between the architectures of cryptic transcription units and *lys2-128*∂. Where examined, Spt^-^ mutants that suppress *lys2-128*∂ allow generation of a truncated transcript that presumably arises from initiation within the Ty1 ∂ element downstream of the *LYS2* promoter. Because the upstream *LYS2* promoter is highly active at *lys2-128*∂, the TSS used in Spt^-^ cells is located within transcribed chromatin, just as cryptic promoters are. For many cryptic transcription-allowing mutants, derepression of cryptic promoters requires disruption of chromatin by transcription (Cheung *et al.* 2008). In other compound transcription units, such as *SRG1-SER3*, transcription over a downstream promoter is required for repression of that promoter, and loss of transcription itself can be sufficient in otherwise wild-type cells to allow promoter activation (Martens *et al.* 2004). Additionally, the *lys2-128*∂ Ty ∂ element is distinct from cryptic promoters in that the ∂ element contains a *bona fide* promoter (Elder *et al.* 1983), which in a full-length Ty1 element would be transcribed. Therefore, we sought to determine the requirement for transcription from the *LYS2* promoter for transcription originating within the Ty1 ∂. We deleted the *LYS2* promoter in three conformations in *SPT6* and *spt6-1004* backgrounds to determine the requirements for *LYS2* transcription in silencing the Ty1 ∂ element or as a condition for the Spt^-^ phenotype in *spt6-1004* or Pol II mutants (Figure 10).

Deletion of the *LYS2* promoter was coupled to the insertion of a marker cassette containing *hphNT1* in either orientation by homologous recombination, or replacement of the *LYS2* promoter with the *GAL1* promoter linked to *kamMX4* to allow ectopic activation in the presence of galactose (see *Materials and Methods*). We obtained the following results (Figure 10): first, deletion of the *LYS2* promoter in multiple conformations does not itself allow activation of the Ty1 ∂ promoter in a fashion that confers a Lys^+^ phenotype, suggesting that *LYS2* transcription does not silence the ∂ promoter in the *lys2-128*∂ context. This result is consistent with prior work suggesting that Ty1 expression requires sequences with the body of the Ty1 element, though transcription was not directly measured in that work (Yu and Elder 1989). Second, deletion of the *LYS2* promoter blocks *spt6-1004* suppression of *lys2-128*∂, suggesting that transcription over the ∂ from *LYS2* is required for the Spt^-^ phenotype. Pol II Spt^-^ mutants were similarly blocked for *lys2-128*∂ suppression in the *GAL1p*::*lys2-128*∂ conformation when cells were grown in glucose. Third, ectopic transcription of *lys2-128*∂ by the *GAL1* promoter in the presence of galactose allowed a weak Lys^+^ phenotype itself, which could be further augmented by either *spt6-1004* or Pol II GOF Spt^-^ mutants. These results suggest that *lys2-128*∂ remains sensitive to Spt^-^-conferring mutants, even when presumed high-level transcription allows a Lys^+^ phenotype in an Spt^+^ background. The ectopic Lys^+^ phenotype for *GAL1p*::*lys2-128*∂ was similarly sensitive to Pol II activity (Figure 10B) as Pol II LOF mutants suppressed it. Thus, suppression of the lys2-128∂ transcription by Pol II LOF extends to both a number of genetic backgrounds and promoter configurations. These results suggest that transcription establishes a state of *lys2-128*∂ that sensitizes it to Spt^-^ mutants for derepression, while remaining sensitive to reduced (LOF) Pol II mutants. Interestingly, we also observed that the Spt^-^ phenotype of *spt6-1004* is weakened in the presence of galactose, while one orientation of *hphNT1* confers a galactose-dependent *spt6-1004* Spt^-^ phenotype. We note that the coding region of *hphNT1* contains at least one possible Gal4 binding site (5′-CGG-N11-CCG-3′) and we speculate this provides ectopic galactose regulation to *lys2-128*∂ that still requires *spt6-1004* for a Lys^+^ phenotype.

### Molecular basis for Pol II mutant effects at lys2-128∂

**Figure 11:**
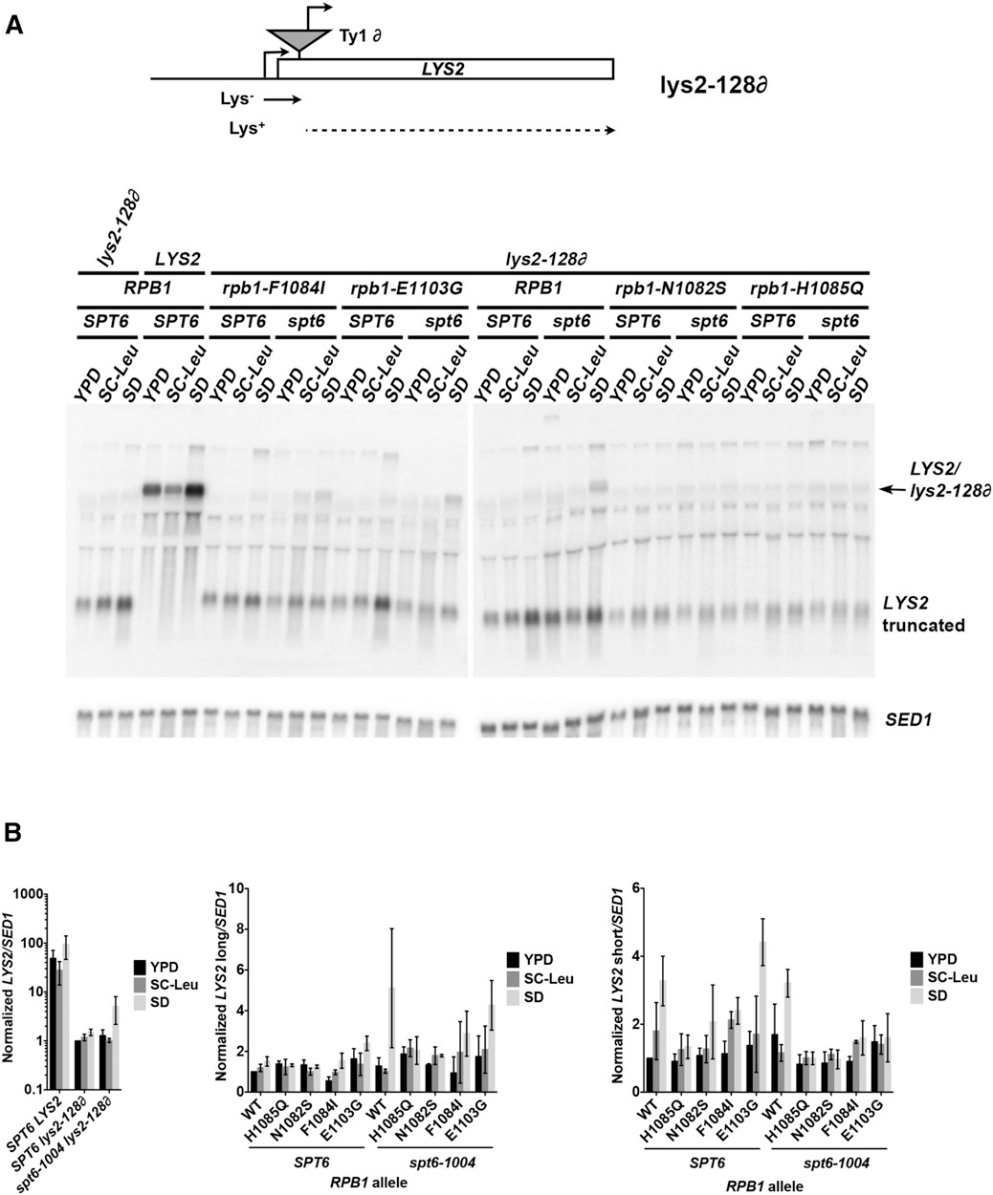
Expression levels of *LYS2* and *lys2-128*∂ transcripts in Spt^-^ phenotype-modulating mutants. (A) Schematic of *lys2-128*∂ (top). Northern blotting for *LYS2/lys2-128*∂-derived RNAs under different media conditions in *rpo21/rpb1* and/or *spt6-1004* (*spt6)* mutant strains. *SED1* mRNA provides a normalization control. (B) Quantification of *LYS2/lys2-128*∂-derived RNA levels under different media conditions in *rpo21/rpb1* and/or *spt6* mutant strains. Error bars indicate standard deviation of the mean of three independent biological replicates. Left: full- or near-full-length *LYS2* mRNA levels normalized to level in WT strain grown in SD comparing *LYS2* with *lys2-128*∂ and effects of *spt6-1004*. Note that scale of *y*-axis is logarithmic. Center: effects on full- or near-full-length *LYS2* mRNA levels in *rpo21/rpb1* and/or *spt6* mutant strains. Right: Effects on truncated (short) *LYS2* mRNA levels in *rpo21/rpb1* and/or *spt6* mutant strains. SD, synthetic defined; WT, wild-type; YPD, yeast extract peptone dextrose.

**Figure 12:**
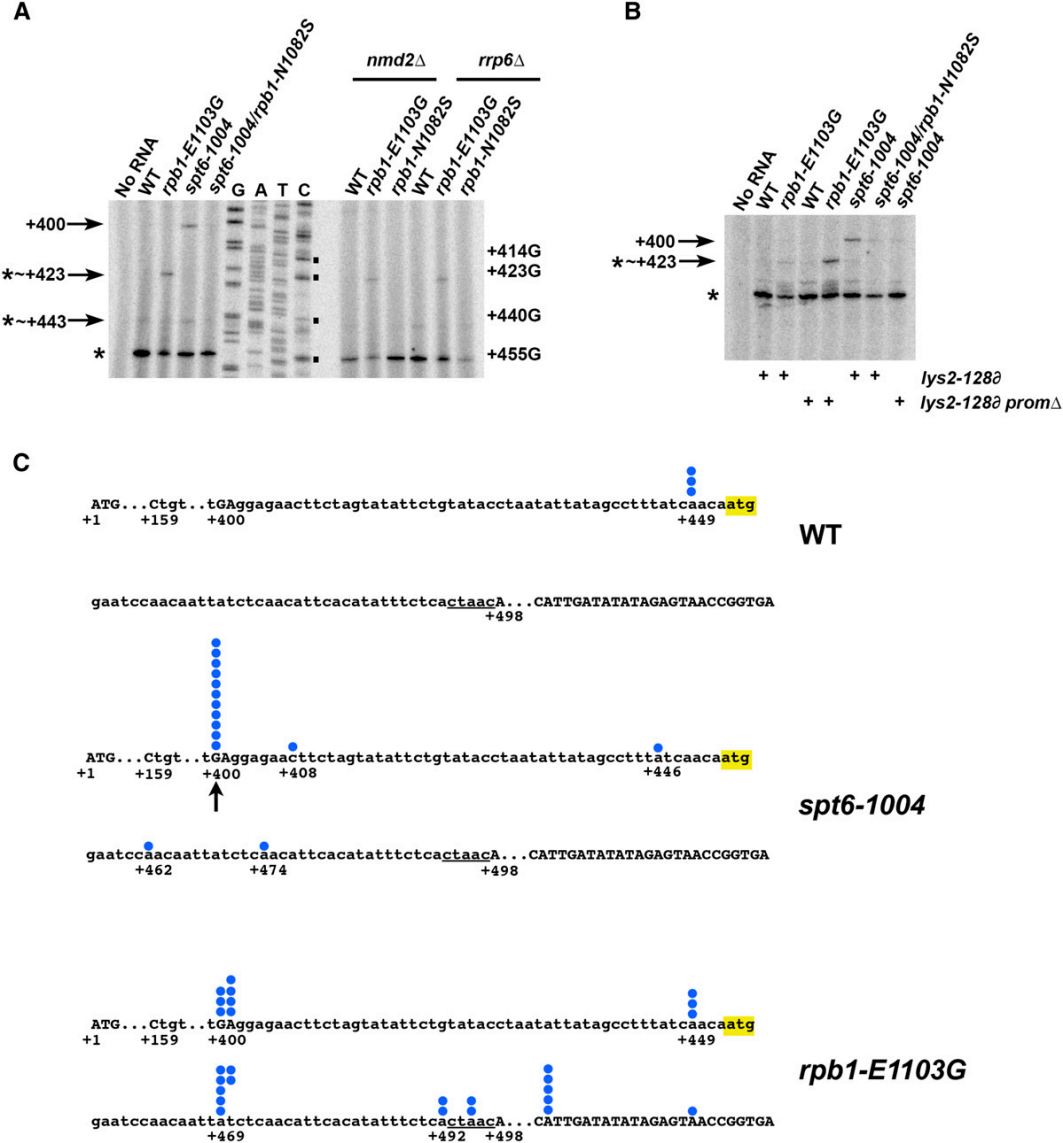
Detection of *lys2-128*∂ transcript 5′ ends in an Spt^-^ Pol II allele *rpb1-E1103G* and *spt6-1004*. (A) Primer extension analysis detecting transcripts emanating from the Ty1 ∂ element at *lys2-128*∂ for *rpo21/rpb1* and *spt6-1004* strains (left). Right side of panel shows *nmd2*∆ and *rrp6*∆ versions of strains for detection of possible unstable transcripts in the region targeted by nonsense-mediated decay (*nmd2*∆) or by the nuclear exosome (*rrp6*∆). Numbered asterisks are bands more apparent in mutant strains but are not specific to *LYS2* (see B). Asterisk indicates nonspecific band in all strains, present even upon deletion of *LYS2* (not shown) “G A T C” represents Sanger DNA sequencing reactions using same primer as primer extension reactions, for identification of putative *lys2-128*∂ transcripts. (B) Primer extension analysis for strains with or without the *LYS2* promoter driving *lys2-128*∂. Deletion of the *LYS2* promoter (*spt6*-1004 strain) or inhibition of transcription upstream of *lys2-128*∂ (*GAL1p:lys2-128*∂, *rpb1-E1103G* strain under glucose growth) suppresses the Spt^-^ phenotypes of *spt6-1004* and *rpo21/rpb1* alleles (Figure 10) and is predicted to abolish transcripts conferring Lys2 function. (C) Schematic showing RNA 5′ ends emanating from the downstream end of the Ty1 ∂ element from *lys2-128*∂ detected by 5′ RACE in WT, *spt6-1004*, and *rpo21/rpb1-E1103G* strains. The +1 position is defined as the A in the *LYS2* ATG. Lower case letters indicate ∂ element sequence. Yellow highlighting indicates ∂ ATG in-frame with downstream *LYS2* sequence. Underlined sequence is a 5 bp duplication created upon Ty1 retrotransposition (Farabaugh and Fink 1980). Blue dots indicate number of clones detected by sequencing and the positions of their 5′ ends. RACE, rapid amplification of cDNA ends; WT, wild-type.

To understand how Spt^-^ mutants suppress *lys2-128*∂ and are themselves suppressed by reduction in Pol II activity, we performed northern blotting for *LYS2* (Figure 11), sequenced *lys2-128*∂ to confirm the location of the ∂ element in *LYS2* and its sequence (Figure S2), performed primer extension and 5′ RACE to determine the transcription start sites for ∂-originating transcripts (Figure 12), and performed 3′ RACE to determine the 3′ termini of *LYS2*-originating transcripts (Figure S3). Northern blotting revealed a detectable increase in an almost full-length *LYS2* transcript for *spt6-1004* (Figure 11A), as previously observed for particular alleles of *SPT4*, *SPT5*, and *SPT6* (Clark-Adams and Winston 1987; Swanson *et al.* 1991; Malone *et al.* 1993). This increase was, however, only a fraction of WT *LYS2* levels (quantitated on the left, Figure 11B), consistent with only a very slight level of expression of truncated *lys2-128*∂ mRNA being needed for a Lys^+^ phenotype. GOF Pol II Spt^-^ mutant E1103G shows a slight increase in *lys2-128*∂ mRNA above WT, however, the Spt^-^ Pol II mutant F1084I does not to a significant extent (Figure 11B). LOF Pol II mutants reduce *lys2-128*∂ mRNA to WT levels in the *spt6-1004* background and do not show the increase in minimal defined (SD) medium over minimal complete (SC-Leu) that was apparent in *spt6-1004* alone, consistent with observations that they can suppress Spt^-^ phenotypes of Spt mutants. When we quantified levels of transcription from the *LYS2* promoter (right, Figure 11B), which terminates within the ∂ element (Figure S3), we found that the Pol II LOF mutant H1085Q blunted the induction of *LYS2* seen in SD medium relative to YPD in WT cells. In the *spt6-1004* background, all Pol II mutants appeared to reduce this induction relative to *spt6-1004* alone, regardless of their Spt^-^ phenotypes. These results suggest that reduction in *LYS2* expression from the upstream *LYS2* promoter alone does not confer reduced Lys^+^ phenotypes or reduced *lys2-128*∂ mRNA expression.

Primer extension analysis revealed that the *spt6-1004* Spt^-^ phenotype likely arises from activation of the normal Ty1 TSS (Elder *et al.* 1983), as primer extension and 5′ RACE (Figure 12, A and B) both identify transcripts consistent with the previously determined Ty1 TSS. This TSS is upstream of an ATG within the ∂ element that is in-frame with the *LYS2* coding sequence. This particular ATG is the only nominal start codon for *LYS2* downstream of the ∂ insertion for hundreds of nucleotides. No transcripts upstream of this ATG were identified for WT cells by primer extension (Figure 12A), while 5′ RACE identified three cDNA clones starting four nucleotides upstream of this ATG from a WT strain, unlikely to allow efficient translation (Figure 12B). Consistent with suppression of Spt^-^ phenotypes by reduced Pol II activity, the *lys2-128*∂ transcript observed by primer extension for *spt6-1004* was suppressed by Pol II N1082S (Figure 12A). For the Spt^-^ Pol II allele *rpb1-E1103G*, we observed a transcript with a presumptive RNA 5′ end within the ∂ element by primer extension (Figure 12A). Given that primer extension can be prone to cross-priming of unrelated transcripts, we ascertained the requirement for the intact *LYS2* promoter, which is required for the Spt^-^ phenotype, of *spt6-1004* and *rpb1-E1103G* transcripts observed by primer extension (Figure 12C). The presumptive ∂ TSS observed by primer extension for *spt6-1004* is abolished upon deletion of the *LYS2* promoter, while another 5′ end seen for all genotypes, and one 5′ end specific to *rpb1-E1103G* at ∼+423 from the *LYS2* ATG, are not affected by deletion/repression of the *LYS2* promoter. Consistent with these observations, we do not observe a presumptive TSS for *rpb1-E1103G* 5′ RACE matching the spurious transcript ∼+423 seen by PE. We conclude that the observed ∼+423 *rpb1-E1103G* transcript is derived from *rpb1-E1103G* effects on an unrelated promoter. However, 5′ RACE did identify transcripts originating from the normal Ty1 TSS and one nucleotide downstream within the ∂ for *rpb1-E1103G*, along with a number of additional putative TSSs unobserved in either *spt6-1004* 5′ RACE or WT 5′ RACE. We speculated that *rpb1-E1103G* might be causing upstream TSS shifts from a cryptic promoter that does not normally generate stable transcripts, just as it shifts TSS upstream at a number of promoters (Kaplan *et al.* 2012). Such transcripts initiating downstream of the ∂ ATG might be unstable due to a lack of in-frame ATG or other reasons. Therefore, we performed primer extension in *nmd2*∆ (defective for Nonsense Mediated Decay) and *rrp6*∆ backgrounds (Figure 12C). We were unable to detect cryptic, unstable transcription in the vicinity of the ∂, but interpretation was hampered by the apparent low level of RNA required for the Spt^-^ phenotype. Finally, we also examined RNA 3′ ends terminating within the ∂ element, as *LYS2* transcription impacts Spt^-^ phenotypes, and it is possible that differential polymerase read-through from the upstream *LYS2* promoter might underlie Pol II mutant effects at *lys2-128*∂, especially in light of known effects of Pol II or Spt mutants on 3′ end formation or termination (Cui and Denis 2003; Kaplan *et al.* 2005; Hazelbaker *et al.* 2013). We did not observe substantial changes in polyadenylation sites within the ∂ in the examined strains (Figure S3). We cannot rule out additional effects on terminating polymerases downstream of the polyadenylation sites.

## Discussion

Determination of the molecular basis for alterations in gene expression brought about by genetic perturbation to transcription or chromatin regulators allows us to understand the orchestration of gene expression and its regulation by chromatin structure. We know now that transcription units can be complex, with promoters adjacent to one another, possibly driving overlapping or antisense transcription. Interestingly, loci in yeast harboring compound transcription units are the bases for a number of *in vivo* growth phenotypes, and these have successfully been exploited for the study of transcriptional processes. These loci share the attribute that transcription may be delicately poised between states allowing for sensitive detection of transcription defects, even for factors functioning in transcription genome-wide and not simply at the loci in question. One such locus in *S. cerevisiae*, *lys2-128*∂, has played an important role in identifying and characterizing conserved transcriptional regulator genes such as *SPT4*, *SPT5*, and *SPT6*, among others. In this work, we addressed genetic relationships between the Spt elongation factors Spt4, Spt5, and Spt6, Pol II catalytic activity, and the Lys^+^ Spt^-^ phenotype conferred by Spt^-^ alleles of Spt factors and Pol II.

We found extensive allele-specific genetic interactions between Spt factors and mutants known to alter Pol II activity. Similar to our previous genetic interaction studies with Pol II alleles and a large panel of gene deletions (Braberg *et al.* 2013), or with viable alleles of essential general transcription factor genes (Jin and Kaplan 2014), we found that genetic interactions were generally dependent on whether Pol II alleles were associated with decreased (LOF) or increased (GOF) Pol II activity. While both classes can have wide-ranging genetic interactions, interactions with particular genes/alleles tend to be stronger with one class or another. All Spt gene alleles tended to show stronger interactions with LOF Pol II alleles, consistent with defects in positive transcription functions showing synergy with a decrease in Pol II catalytic rate. The greater allele-specificity between *spt4*∆*/spt5-194* and Pol II LOF alleles than for *spt6-1004* are consistent with direct positive roles of the Spt4/Spt5 complex in elongation, stemming from its likely close association with the Pol II elongation complex (Wada *et al.* 1998; Bourgeois *et al.* 2002; Zhang *et al.* 2004; Hartzog and Kaplan 2011; Martinez-Rucobo *et al.* 2011).

Interactions between Pol II catalytic rate alleles and *spt5-242* indicate complexities about which we might only speculate. Previously, suppressors of *spt5-242* have been interpreted as being of two classes (Hartzog *et al.* 1998; Quan and Hartzog 2010). The first class alters Pol II function such that it is slower, while the second class alters chromatin structure such that transcribed chromatin is expected to be disrupted. The former is proposed to allow Pol II additional time to deal with obstacles to transcription in light of defective Spt5 function in *spt5-242*, while the latter class was proposed to reduce chromatin obstacles to Pol II elongation, alleviating some requirements for WT Spt5 function. Our observations here show that many Pol II mutants with *bona fide* catalytic defects suppress *spt5-242* regardless of LOF or GOF status. Furthermore, most Pol II alleles, whether presumed fast or slow, enhance *spt5-242* growth defects at 37°. These results are consistent with the previously proposed model that Pol II activity and Spt5 function need to be kinetically matched for proper transcription (Hartzog *et al.* 1998). Additionally, it is possible that increase in Pol II activity allows some elongation barriers to be bypassed, also alleviating *spt5-242* defects. Further consideration of these issues will require direct evidence that elongation phenotypes of Pol II alleles are apparent *in vivo* as predicted from *in vitro* elongation rates (Malagon *et al.* 2006; Kaplan *et al.* 2008, 2012; Kireeva *et al.* 2008; Larson *et al.* 2012). Human analogs of some Pol II mutants assessed here (*rpb1-E1103G* and *rpb1-H1085Y*) have been shown to be fast or slow in a human cell line (Fong *et al.* 2014), as predicted from previous biochemical experiments on yeast Pol II mutant enzymes. One of the Pol II mutants analyzed here, *rpb1-E1103G*, was reported to elongate faster than WT *in vivo* as predicted (Hazelbaker *et al.* 2013); however, we are currently revisiting this conclusion.

From the results presented here, we hypothesize that Pol II alleles suppress *lys2-128*∂ for reasons distinct from Spt elongation factor alleles, or at a minimum distinct from *spt6-1004*. We find that *lys2-128*∂ has features common to cryptic intragenic transcription, including a requirement for transcription from the *LYS2* promoter for the *spt6-1004* Spt^-^ phenotype at *lys2-128*∂. Coupled with previous work suggesting that chromatin proximal to Ty1 or Ty1 ∂ elements in the yeast genome is sensitive to *spt6-1004* (Perales *et al.* 2013), *spt6-1004* activation of *lys2-128*∂ likely proceeds through *LYS2* promoter-dependent transcriptional disruption of nucleosomes that repress the *lys2-128*∂ promoter. We do not favor a similar mechanism for Pol II Spt alleles’ Spt^-^ phenotypes, though our conclusions are necessarily tempered by the low level of *lys2-128*∂ expression underlying their phenotypes. We argue as follows: first, we would expect synthetic or enhancing interactions between Pol II Spt^-^ alleles and Spt elongation factors if their respective defects were in similar functions. Second, we might expect to a greater modulation of cryptic transcription by Pol II alleles than we have observed, if altered Pol II activity suppressed *lys2-128*∂ through elongation-mediated chromatin disruption. Though activation of a cryptic transcription reporter can be observed for one of the most extreme Pol II mutants, *rpb1-G1097D*, suppression is weak. The identification of a number of 5′ RACE clones indicating potential TSSs in *rpb1-E1103G* downstream of the Ty1 ∂ TSS raises the possibility that *rpb1-E1103G* initiation and not elongation defects underlie its Spt^-^ phenotype. For example, *rpb1-E1103G* might activate a cryptic promoter by allowing initiation from positions upstream of “frustrated” initiation sites where initiation is abortive or nonproductive. A prediction of this model is that mutants that generally shift TSSs upstream at promoters should confer the Spt^-^ phenotype, and that mutants that can suppress upstream TSS shifts in *rpb1-E1103G* should suppress this phenotype. In support of these predictions, we showed previously that a *tfg2* allele (in Pol II initiation factor TFIIF) confers a weak Spt^-^ phenotype along with shifting TSSs upstream at analyzed promoters (*e.g.*, *ADH1*) (Jin and Kaplan 2014). Furthermore, Pol II Spt^-^ alleles’ Spt phenotype at *lys2-128*∂ was suppressed by alleles of *SUA7*, encoding yeast TFIIB. These *sua7* alleles shift TSSs downstream on their own, and suppress upstream TSS shifts of Pol II alleles at *ADH1* (Jin and Kaplan 2014). Taken together, these results suggest that the interplay of Pol II initiation and elongation can converge at *lys2-128*∂ to render transcription finely balanced between nonfunctional and functional *LYS2* expression.

## Supplementary Material

Supplemental Material
