# Supplementary material for: Relationships Between RNA Polymerase II Activity and Spt Elongation Factors to Spt- Phenotype and Growth in Saccharomyces cerevisiae
Source: G3 (Bethesda). 2016 Jun 3;6(8):2489–504. doi: 10.1534/g3.116.030346 (PMC4978902; doi:10.1534/g3.116.030346)
Supplement: Supplemental Material [file supp_6_8_2489__index.html]

Relationships Between RNA Polymerase II Activity and Spt Elongation Factors to Spt- Phenotype and Growth in Saccharomyces cerevisiae — Supplemental Material 

# Relationships Between RNA Polymerase II Activity and Spt Elongation Factors to Spt- Phenotype and Growth in *Saccharomyces cerevisiae*

## Supplemental Material for Cui *et al.*, 2016

**Files in this Data Supplement:**

- Figure S1 - Dominant phenotypes of *rpo21/rpb1* plasmids in the *spt6-1004* background. (.pdf, 292 KB)
- Figure S2 - Sequence of lys2-128δ. (.pdf, 144 KB)
- Figure S3 - 3' RACE analysis at lys2-128δ. (.pdf, 192 KB)
- Table S1 - Yeast strains. (.pdf, 105 KB)
- Table S2 - Plasmids. (.pdf, 126 KB)
- Table S3 - Oligo sequences. (.pdf, 101 KB)
- File S1 - Supplemental literature cited. (.pdf, 60 KB)
