# Supplementary material for: Relationships Between RNA Polymerase II Activity and Spt Elongation Factors to Spt- Phenotype and Growth in Saccharomyces cerevisiae
Source: G3 (Bethesda). 2016 Jun 3;6(8):2489–504. doi: 10.1534/g3.116.030346 (PMC4978902; doi:10.1534/g3.116.030346)
Supplement: Supplemental Material [file supp_g3.116.030346_FigureS1.pdf]

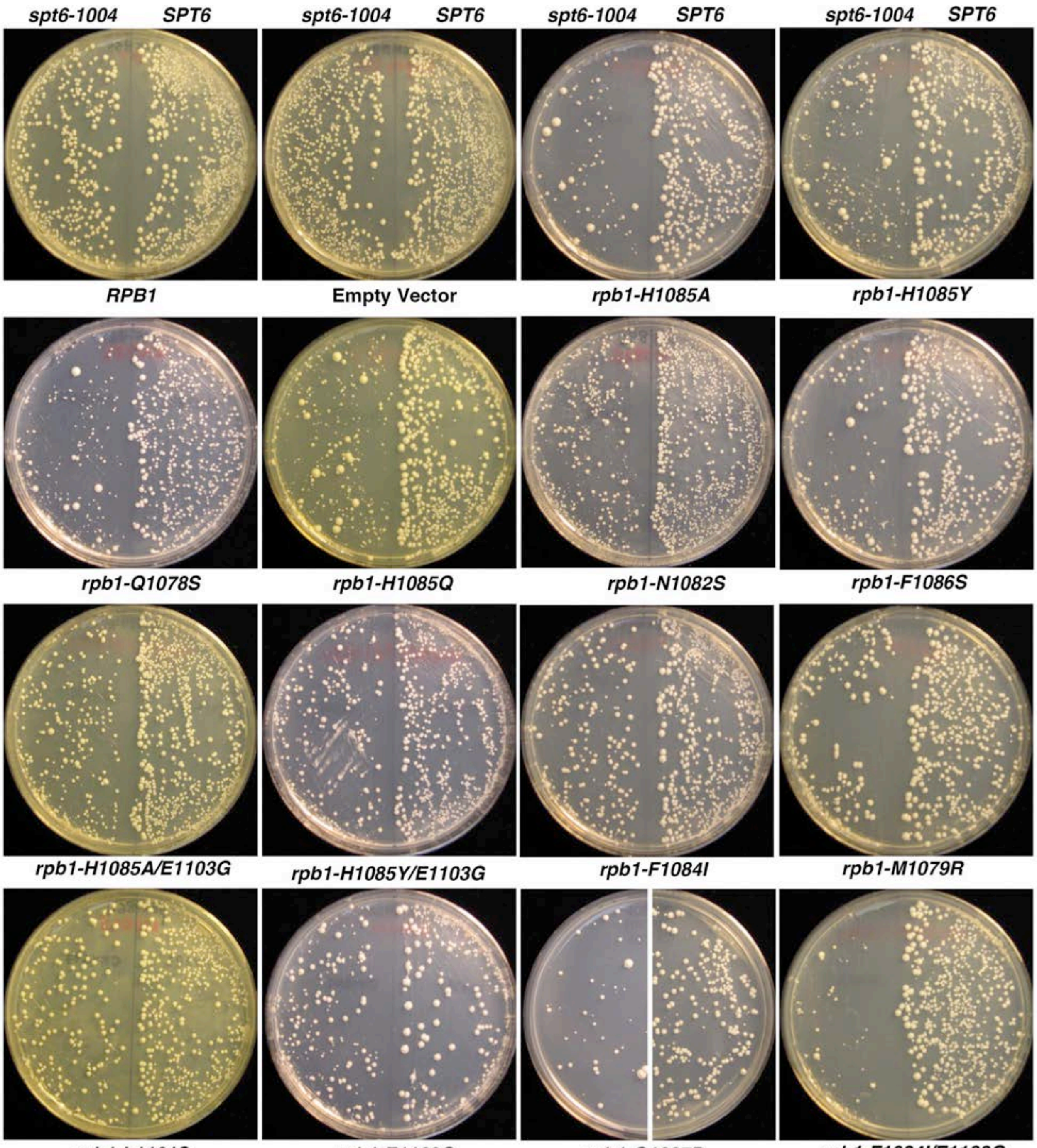

Figure S1. Dominant phenotypes of *rpo21/rpb1* plasmids in the *spt6-1004* background. Yeast plates for CKY697 (containing *spt6-1004*, *rpo21*Δ, [*RPO21/RPB1 CEN URA3*]) or CKY283 (*SPT6*, *rpo21*Δ*,* [*RPO21/RPB1 CEN URA3*]) showing Leu$^+$ transformants after transformation with [*rpo21/rpb1* mutant *CEN LEU2*] or control *CEN LEU2* plasmids.
