# Supplementary material for: Relationships Between RNA Polymerase II Activity and Spt Elongation Factors to Spt- Phenotype and Growth in Saccharomyces cerevisiae
Source: G3 (Bethesda). 2016 Jun 3;6(8):2489–504. doi: 10.1534/g3.116.030346 (PMC4978902; doi:10.1534/g3.116.030346)
Supplement: Supplemental Material [file supp_g3.116.030346_FigureS2.pdf]

+1
ATGACTAACGAAAAGGTCTGGATAGAGAAGTTGGATAATCCAACTCTTTCAGTGTTACCACATGACTTTTTACGCCCACA

+159
ACAAGAACCTTATACGAAACAAGCTACATATTCGTTACAGCTACCTCAGCTCGATGTGCCTCATGATAGTTTTTCTAA**C**t

gttggaatagaaatcaactatcatctactaactagtatttacattactagtatattatcatatacggtgttagaagatga

cgcaaatgatgagaaatagtcatctaaattagtggaagctgaaacgcaaggattgataatgtaataggatcaatgaatat

+400
aaacatataaaatgatgataataatatttatagaattgtgtagaattgcagattccctttatggattcctaaatcctt**G**

+453
aggagaacttctagtatattctgtatacctaatattatagcctttatcaaca**a**tggaatccaacaattatctcaacattc

+498
acatatttctcactaac**A**AATACGCTGTCGCTTTGAGTGT
+160

Figure S2. Sequence of lys2-128∂. The +1 position is defined as the A in the *LYS2* ATG. Lower case letters indicate Ty1 ∂ element sequence. Yellow highlighting indicates ∂ ATG in frame with downstream *LYS2* sequence. Bolded "G" at +400 is the vicinity of the Ty1 TSS. Underlined sequence is 5 base-pair duplication created upon Ty1 retrotransposition (FARABAUGH and FINK 1980). The ∂ insertion is between *LYS2* position +159 and +160
