# Supplementary material for: Relationships Between RNA Polymerase II Activity and Spt Elongation Factors to Spt- Phenotype and Growth in Saccharomyces cerevisiae
Source: G3 (Bethesda). 2016 Jun 3;6(8):2489–504. doi: 10.1534/g3.116.030346 (PMC4978902; doi:10.1534/g3.116.030346)
Supplement: Supplemental Material [file supp_g3.116.030346_FigureS3.pdf]

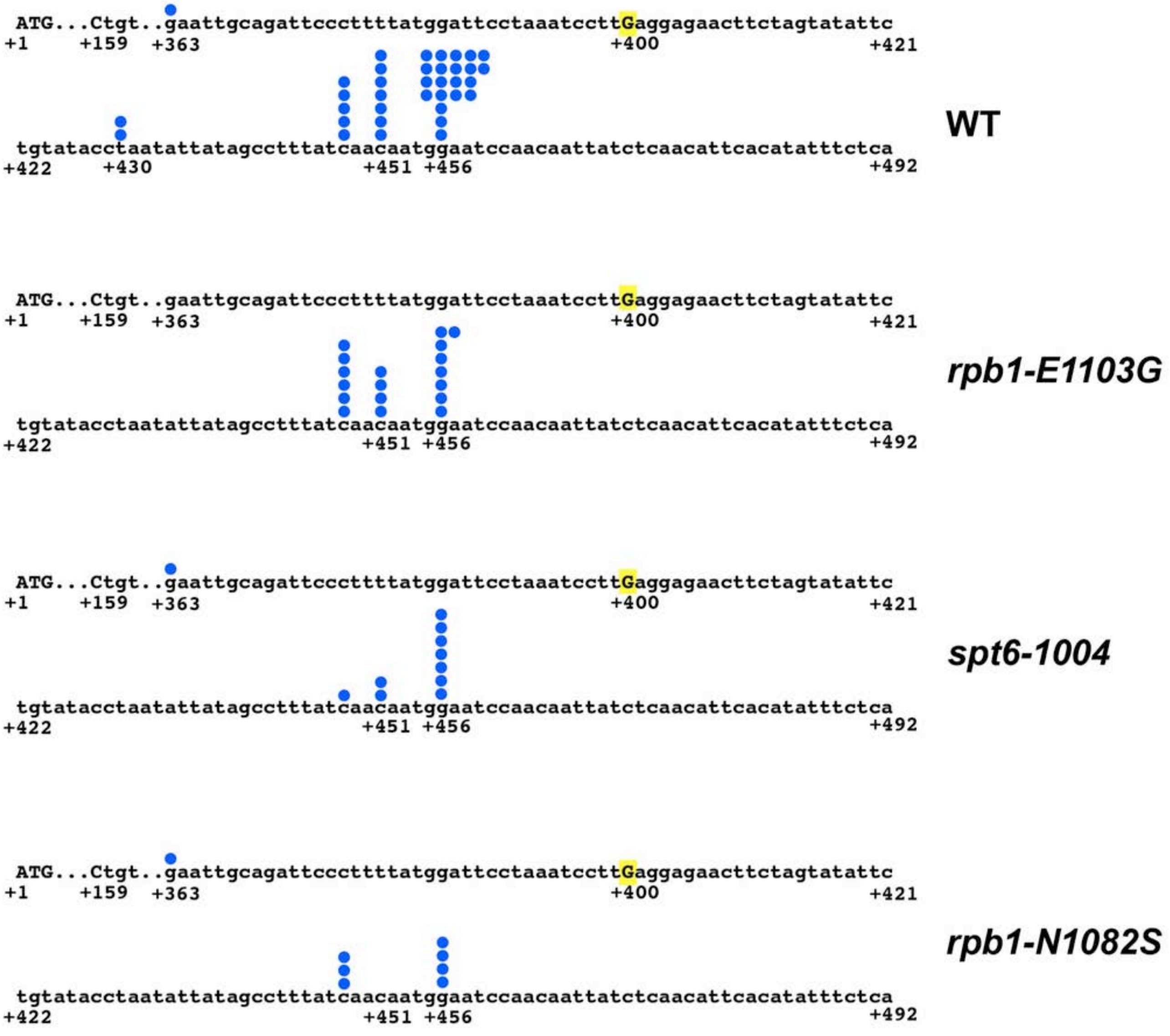

Figure S3. 3′ RACE analysis at *lys2-128∂*. *LYS2* ATG "A" is designated +1. +400 Ty1 ∂ TSS observed in 5′ RACE is highlighted yellow. Blue dots indicate 3′ ends identified by sequencing of cloned cDNAs for particular strains (relevant genotypes noted on right).
