# Supplementary material for: Relationships Between RNA Polymerase II Activity and Spt Elongation Factors to Spt- Phenotype and Growth in Saccharomyces cerevisiae
Source: G3 (Bethesda). 2016 Jun 3;6(8):2489–504. doi: 10.1534/g3.116.030346 (PMC4978902; doi:10.1534/g3.116.030346)
Supplement: Supplemental Material [file supp_g3.116.030346_TableS1.pdf]

Table S1 Yeast Strains

Strain Number	Genotype	Alias	Source	Derived from	Figure
CKY283	*MAT***a** *ura3-52 his3Δ200 leu2Δ1 or Δ0 trp1Δ63 met15Δ0 lys2-128∂ gal10Δ56 rpb1Δ::NATMX4 RPB3::TAP::KlacTRP1* [*pRP112 CEN URA3 RPB1*]		Kaplan Lab		1-12, S3
CKY697	*MAT***a** *ura3-52 his3Δ200 leu2Δ1 or Δ0 trp1Δ63 met15Δ0 lys2-128∂ gal10Δ56 rpb1Δ::NATMX4 spt6-1004 RPB3::TAP::KlacTRP1* [*pRP112 CEN URA3 RPB1*]		Kaplan Lab		2,5,6,9-12, S3
GHY339	*MATalpha his4-912∂ his3Δ200 or HIS3 lys2-128∂ leu2Δ1 ura3-52 spt5-242 rpb1Δ187::HIS3* [pRP112 *CEN URA3 RPB1*]	CKY660	Grant Hartzog		1,4
GHY627	*MATalpha his4-912∂ his3Δ200 or HIS3 lys2-128∂ leu2Δ1 trp1Δ63 ura3-52 sptΔ2::HIS3 rpb1Δ187::HIS3* [*pRP112 CEN URA3 RPB1*]	CKY661	Grant Hartzog		1,3
GHY949	*MATa his3d200 lys2-128∂ leu2d1 ura3-52 trp1d63 spt5-194 rpb1Δ187::HIS3* [*pRP112 CEN URA3 RPB1*]	CKY662	Grant Hartzog		1,3
CKY1078-CKY1081 **CKY1079**	*MAT***a** *ura3-52 his3Δ200 leu2Δ1 or Δ0 trp1Δ63 met15Δ0 lys2-128∂ gal10Δ56 rpb1Δ::NATMX4 RPB3::TAP::KlacTRP1 LYS2pΔ::hphNT1::lys2-128∂ (Orientation 1)* [*pRP112 CEN URA3 RPB1*]		Kaplan Lab	CKY283	10
CKY1082-CKY1085 **CKY1083**	*MAT***a** *ura3-52 his3Δ200 leu2Δ1 or Δ0 trp1Δ63 met15Δ0 lys2-128∂ gal10Δ56 rpb1Δ::NATMX4 RPB3::TAP::KlacTRP1 LYS2pΔ::hphNT1::lys2-128∂ (Orientation 2)* [*pRP112 CEN URA3 RPB1*]		Kaplan Lab	CKY283	10,12
CKY1074-CKY1077 **CKY1075**	*MAT***a** *ura3-52 his3Δ200 leu2Δ1 or Δ0 trp1Δ63 met15Δ0 lys2-128∂ gal10Δ56 rpb1Δ::NATMX4 RPB3::TAP::KlacTRP1 kanMX4::GAL1p::lys2-128∂* [*pRP112 CEN URA3 RPB1*]		Kaplan Lab	CKY283	10
CKY1090-CKY1093 **CKY1091**	*MAT***a** *ura3-52 his3Δ200 leu2Δ1 or Δ0 trp1Δ63 met15Δ0 lys2-128∂ gal10Δ56 rpb1Δ::NATMX4 spt6-1004 RPB3::TAP::KlacTRP1 LYS2pΔ::hphNT1::lys2-128∂ (Orientation 1)* [*pRP112 CEN URA3 RPB1*]		Kaplan Lab	CKY283	10
CKY1094-CKY1097 **CKY1095**	*MAT***a** *ura3-52 his3Δ200 leu2Δ1 or Δ0 trp1Δ63 met15Δ0 lys2-128∂ gal10Δ56 rpb1Δ::NATMX4 spt6-1004 RPB3::TAP::KlacTRP1 LYS2pΔ::hphNT1::lys2-128∂ (Orientation 2)* [*pRP112 CEN URA3 RPB1*]		Kaplan Lab	CKY283	10,12
CKY1086-CKY1089 **CKY1087**	*MAT***a** *ura3-52 his3Δ200 leu2Δ1 or Δ0 trp1Δ63 met15Δ0 lys2-128∂ gal10Δ56 rpb1Δ::NATMX4 spt6-1004 RPB3::TAP::KlacTRP1 kanMX4::GAL1p::lys2-128∂* [*pRP112 CEN URA3 RPB1*]		Kaplan Lab	CKY283	10
FY603	*MATalpha leu2Δ1 ura3-52 trp1Δ63 his3Δ200 lys2-128∂*	CKY458	Fred Winston		10
CKY1106-CKY1109 **CKY1107**	*MATalpha leu2Δ1 ura3-52 trp1Δ63 his3Δ200 lys2-128∂ LYS2pΔ::hphNT1::lys2-128∂ (Orientation 1)*		Kaplan Lab	CKY458	10
CKY1102-CKY1105 **CKY1103**	*MATalpha leu2Δ1 ura3-52 trp1Δ63 his3Δ200 lys2-128∂ LYS2pΔ::hphNT1::lys2-128∂ (Orientation 2)*		Kaplan Lab	CKY458	10
CKY1098-CKY1101 **CKY1099**	*MATalpha leu2Δ1 ura3-52 trp1Δ63 his3Δ200 lys2-128∂ kanMX4::GAL1p::lys2-128∂*		Kaplan Lab	CKY458	10
CKY1167	*MATalpha leu2Δ1 ura3-52 trp1Δ63 his3Δ200 lys2-128∂ spt6-1004*		Kaplan Lab		10

CKY1171	*MATalpha leu2Δ1 ura3-52 trp1Δ63 his3Δ200 lys2-128∂ spt6-1004 LYS2pΔ::hphNT1::lys2-128∂ (Orientation 1)*	Kaplan Lab	CKY1166/ CKY1167	10
CKY1169	*MATalpha leu2Δ1 ura3-52 trp1Δ63 his3Δ200 lys2-128∂ spt6-1004 LYS2pΔ::hphNT1::lys2-128∂ (Orientation 2)*	Kaplan Lab	CKY1166/ CKY1167	10
CKY1173	*MATalpha leu2Δ1 ura3-52 trp1Δ63 his3Δ200 lys2-128∂ spt6-1004 kanMX4::GAL1p::lys2-128∂*	Kaplan Lab	CKY1166/ CKY1167	10
CKY950	*MAT***a** *lys2-128∂ ura3-52 his3Δ200 leu2Δ0 or Δ1 trp1Δ63 rpb1Δ::natMX4 kanMX4::GAL1p::flo8::HIS3* [*pCK518 CEN URA3 RPB1*]	Kaplan Lab	CKY283 x FY2713 (alias CKY827)	8
CKY952	*MATalpha lys2-128∂ ura3-52 his3Δ200 leu2Δ0 or Δ1 trp1Δ63 rpb1Δ::natMX4 RPB3-TAP::KlTRP1 kanMX4::GAL1p::flo8::HIS3 [pCK518 CEN URA3 RPB1]*	Kaplan Lab	CKY283 x FY2713 (alias CKY827)	8
		Kaplan Lab		8
CKY924	*MATa ura3-52 his3Δ200 leu2Δ1 or Δ0 trp1Δ63 met15Δ0 lys2-128∂ gal10Δ56 rpb1Δ::natMX4 RPB3::TAP::KlacTRP1 rrp6Δ::kanMX4* [*pRP112 CEN URA3 RPB1*]	Kaplan Lab	CKY283	8
CKY715	*MATa ura3-52 his3Δ200 leu2Δ1 or Δ0 trp1Δ63 met15Δ0 lys2-128∂ gal10Δ56 rpb1Δ::NATMX4 RPB3::TAP::KlacTRP1 nmd2Δ::kanMX4* [*pRP112 CEN URA3 RPB1*]	Kaplan Lab	CKY283	8
