# Supplementary material for: Relationships Between RNA Polymerase II Activity and Spt Elongation Factors to Spt- Phenotype and Growth in Saccharomyces cerevisiae
Source: G3 (Bethesda). 2016 Jun 3;6(8):2489–504. doi: 10.1534/g3.116.030346 (PMC4978902; doi:10.1534/g3.116.030346)
Supplement: Supplemental Material [file supp_g3.116.030346_TableS2.pdf]

Table S2 Plasmids

Plasmid	Gene	Allele	Marker/type	Reference
pCK859	*RPO21/RPB1*	WT	*LEU2/CEN*	(KAPLAN *et al.* 2012)
pCK863	*rpo21/rpb1*	Q1078S	*LEU2/CEN*	(KAPLAN *et al.* 2012)
pCK886	*rpo21/rpb1*	N1082S (T1161R)*	*LEU2/CEN*	(KAPLAN *et al.* 2012)
pCK887	*rpo21/rpb1*	H1085Q	*LEU2/CEN*	(KAPLAN *et al.* 2012)
pCK870	*rpo21/rpb1*	H1085Y	*LEU2/CEN*	(KAPLAN *et al.* 2012)
pCK871	*rpo21/rpb1*	F1086S	*LEU2/CEN*	(KAPLAN *et al.* 2012)
pCK890	*rpo21/rpb1*	H1085Y/E1103G	*LEU2/CEN*	(KAPLAN *et al.* 2012)
pCK898	*rpo21/rpb1*	N1082S/E1103G	*LEU2/CEN*	(KAPLAN *et al.* 2012)
pCK872	*rpo21/rpb1*	M1079R	*LEU2/CEN*	(KAPLAN *et al.* 2012)
pCK955	*rpo21/rpb1*	F1084I	*LEU2/CEN*	(KAPLAN *et al.* 2012)
pCK867	*rpo21/rpb1*	G1097D	*LEU2/CEN*	(KAPLAN *et al.* 2012)
pCK864	*rpo21/rpb1*	L1101S	*LEU2/CEN*	(KAPLAN *et al.* 2012)
pCK960	*rpo21/rpb1*	E1103G	*LEU2/CEN*	(KAPLAN *et al.* 2012)
pCK901	*rpo21/rpb1*	H1085Q/E1103G	*LEU2/CEN*	(KAPLAN *et al.* 2012)
pCK1340	*rpo21/rpb1*	N1082S	*LEU2/CEN*	(KAPLAN *et al.* 2012)
pCK856	*rpo21/rpb1*	N479S	*LEU2/CEN*	(KAPLAN *et al.* 2012)
pCK923	*rpo21/rpb1*	D526G	*LEU2/CEN*	(KAPLAN *et al.* 2012)
pCK861	*rpo21/rpb1*	H1085A	*LEU2/CEN*	(KAPLAN *et al.* 2012)
pCK863	*rpo21/rpb1*	Q1078S	*LEU2/CEN*	(KAPLAN *et al.* 2012)
pCK899	*rpo21/rpb1*	H1085A/E1103G	*LEU2/CEN*	(KAPLAN *et al.* 2012)
pCK901	*rpo21/rpb1*	H1085Q/E1103G	*LEU2/CEN*	(KAPLAN *et al.* 2012)
pCK952	*rpo21/rpb1*	F1084I/E1103G	*LEU2/CEN*	(KAPLAN *et al.* 2012)
pRS423	-		*HIS3*/2µ	(CHRISTIANSON *et al.* 1992)
pCK848	*SPT6*		*HIS3*/2µ	This work
pCK125	*spt6*	*spt6-1004*	*URA3*	Winston Lab
pFA6a-hphNT1			hphNT1	(JANKE *et al.* 2004)
pYM-N22			*GAL1* promoter, kanMX4	(JANKE *et al.* 2004)

*As noted in Methods and Materials, Table S1, and Figure S6 of our previous work, (JIN and KAPLAN 2014), we found an additional spurious mutation in pCK886 (nominally N1082S, but also containing T1161R). The strains created in the current work were mostly constructed and analyzed prior to that finding. We examined strains harboring pCK886 (*rpo21/rpb1* N1082S T1161R) compared with those harboring pCK1340 (*rpo21/rpb1* N1082S) and did not identify any differences in growth under a number of conditions.
