# Supplementary material for: Relationships Between RNA Polymerase II Activity and Spt Elongation Factors to Spt- Phenotype and Growth in Saccharomyces cerevisiae
Source: G3 (Bethesda). 2016 Jun 3;6(8):2489–504. doi: 10.1534/g3.116.030346 (PMC4978902; doi:10.1534/g3.116.030346)
Supplement: Supplemental Material [file supp_g3.116.030346_TableS3.pdf]

**Table S3 Oligo sequences**

| CKO Number | Sequence | Function | Gene | Name |
|---|---|---|---|---|
| CKO099 | CCCAGCGTTCCGTAGCAAAGGCGTCAGGATC GTCAAACGTCCTTTTTTTTTTTTTTTTT | 3′ RACE Oligo dT primer | | APdT |
| CKO100 | CCCAGCGTTCCGTAGCAAAGG | 3′ RACE primer | | UAP |
| CKO101 | CGTCAGGATCGTCAAACGTCC | Nested 3′ RACE primer | | AUAP |
| CKO468 | AAGATGAAATTATCAACTGTCC | Northern Probe | *SED1* | SED1 F |
| CKO469 | CATAGCAACACCAGCCAAACC | Northern Probe | *SED1* | SED1 R |
| CKO551 | ATGACTAACGAAAAGGTCTGG | Northern Probe | *LYS2* | LYS2 F |
| CKO552 | CTGGCTTGTCAAATCTTGGG | Northern Probe | *LYS2* | LYS2 MID R |
| CKO105 | TTCGGACTCTTTTACGAGGG | Northern Probe | *FLO8* | FLO8 F |
| CKO106 | ATTGAGATCGTAATCCGGTC | Northern Probe | *FLO8* | FLO8 R |
| CKO553 | GGAACAGACACAAACAGCAG | Northern Probe | *STE11* | STE11 F |
| CKO554 | TCAAATTATGTGTGCATCCAG | Northern Probe | *STE11* | STE11 R |
| CKO843 | TAACGAAAAGGTCTGGATAG | For 3′ RACE of *LYS2* | *LYS2* | LYS2 5′ alt1 |
| CKO844 | GAGAAGTTGGATAATCCAAC | For nested 3′ RACE of *LYS2* (after CKO843) | *LYS2* | LYS2 5′ alt2 |
| CKO839* | CACTTGCAATTACATAAAAAATTCCGGCGGTT TTTCGCGTG**<u>CGTACGCTGCAGGTCGAC</u>** | KO or replacement of *LYS2* promoter | *LYS2* | lys2ΔPROM F |
| CKO840* | TTGGATTATCCAACTTCTCTATCCAGACCTTTT CGTTAGT**<u>CATCGATGAATTCTCTGTCG</u>** | KO or replacement of *LYS2* promoter | *LYS2* | lys2ΔPROM R |
| CKO841* | CACTTGCAATTACATAAAAAATTCCGGCGGTT TTTCGCGTG**<u>ATCGATGAATTCTCTGTCG</u>** | KO of *LYS2* promoter | *LYS2* | lys2ΔPROM_REVORIENT F |
| CKO842* | TTGGATTATCCAACTTCTCTATCCAGACCTTTT CGTTAGT**<u>CGTACGCTGCAGGTCGAC</u>** | KO of *LYS2* promoter only | *LYS2* | lys2ΔPROM_REVORIENT R |
| CKO1569 | TCATTGTTAATTGTAGAATACAGC | lys2-128∂ PE downstream | *LYS2* | lys2-128∂ Primer Extension /5′ RACE |
| CKO1585 | TCATTAAATGACCACGTTGGT | LYS2 RACE CKO1569 NESTED | *LYS2* | lys2-128∂ 5′ RACE NESTED primer |

**<u>*Bold, underline</u>** sequences represent portion of primer directed to plasmid for amplification of marker cassettes.
