# Supplementary material for: Relationships Between RNA Polymerase II Activity and Spt Elongation Factors to Spt- Phenotype and Growth in Saccharomyces cerevisiae
Source: G3 (Bethesda). 2016 Jun 3;6(8):2489–504. doi: 10.1534/g3.116.030346 (PMC4978902; doi:10.1534/g3.116.030346)
Supplement: Supplemental Material [file supp_g3.116.030346_FileS1.pdf]

SUPPLEMENTAL LITERATURE CITED

CHRISTIANSON, T. W., R. S. SIKORSKI, M. DANTE, J. H. SHERO and P. HIETER, 1992 Multifunctional yeast high-copy-number shuttle vectors. Gene **110:** 119-122.

JANKE, C., M. M. MAGIERA, N. RATHFELDER, C. TAXIS, S. REBER *et al.*, 2004 A versatile toolbox for PCR-based tagging of yeast genes: new fluorescent proteins, more markers and promoter substitution cassettes. Yeast **21:** 947-962.

JIN, H., and C. D. KAPLAN, 2014 Relationships of RNA polymerase II genetic interactors to transcription start site usage defects and growth in Saccharomyces cerevisiae. G3 (Bethesda) **5:** 21-33.

KAPLAN, C. D., H. JIN, I. L. ZHANG and A. BELYANIN, 2012 Dissection of Pol II trigger loop function and Pol II activity-dependent control of start site selection in vivo. PLoS Genet **8:** e1002627.
